# 3D nanoprinted fiber-interfaced hollow-core waveguides for high-accuracy nanoparticle tracking analysis

**DOI:** 10.1038/s41377-025-01827-9

**Published:** 2025-05-15

**Authors:** Diana Pereira, Torsten Wieduwilt, Walter Hauswald, Matthias Zeisberger, Marta S. Ferreira, Markus A. Schmidt

**Affiliations:** 1 Leibniz Institute of Photonic Technology, Jena, Germany; 2 i3N & Physics Department, University of Aveiro, Campus de Santiago, Aveiro, Portugal; 3 Abbe Center of Photonics and Faculty of Physics, Friedrich Schiller University Jena, Jena, Germany; 4 Otto Schott Institute of Materials Research (OSIM), Friedrich Schiller University Jena, Jena, Germany

**Keywords:** Integrated optics, Nanoparticles

## Abstract

The integration of functional components into flexible photonic environments is a critical area of research in integrated photonics and is essential for high-precision sensing. This work presents a novel concept of interfacing square-core hollow-core waveguides with commercially available optical fibers using 3D nanoprinting, and demonstrates its practical relevance through a nanoscience-based characterization technique. In detail, this innovative concept results in a monolithic, fully fiber-integrated device with key advantages such as alignment-free operation, high-purity fundamental mode excitation, full polarization control, and a unique handling flexibility. For the first time, the application potential of a fiber-interfaced waveguide in nanoscale analysis is demonstrated by performing nanoparticle-tracking-analysis experiments. These experiments involve the tracking and analysis of individual gold nanospheres diffusing in the hollow core waveguide, enabled by nearly aberration-free imaging, extended observation times, and homogeneous light-line illumination. The study comprehensively covers design strategy, experimental implementation, key principles, optical characterization, and practical applications. The fiber-interfaced hollow-core waveguide concept offers significant potential for applications in bioanalytics, environmental sciences, quantum technologies, optical manipulation, and life sciences. It also paves the way for the development of novel all-fiber devices that exploit enhanced light-matter interaction in a monolithic form suitable for flexible and remote applications.

## Introduction

The integration of functional components in flexible photonic environments is a key research area in integrated photonics and is particularly important for sensing applications, e.g. to reduce sample volumes and geometric device footprints^1^. One promising approach relies on the use of waveguides that guide light into the medium of interest, maximizing light-matter interaction^2^. However, efficient waveguiding in water is challenging because its low refractive index prevents total internal reflection at the liquid/cladding interface, precluding the use of many materials.

A current research direction in photonics that addresses this issue is Hollow Core Waveguides (HCWs), which rely on complex light-guiding mechanisms via nano- and microstructured claddings. Compared to their evanescent counterparts, these waveguides offer several key advantages especially for sensing applications, one example being the close to 100% modal overlap with the medium of interest, allowing direct application of Beer-Lambert’s law without any modification.

The majority of HCWs are found in fiber optics, where significant progress has been made^3^. In addition to photonic bandgap fibers^4^, recent advances in anti-resonant fibers have achieved excellent light-guiding performance with reduced structural complexity^5^. These developments have enabled numerous applications, including nonlinear optics (e.g., UV generation^6^), sensing (e.g., drug detection^7^), and quantum technologies (e.g., excitation of Rydberg states^8^).

Recently, the limited portfolio of on-chip HCWs, primarily based on anti-resonant reflective optical waveguides (ARROWs)^9,10^, has been expanded by translating fiber-optic concepts to planar photonics using 3D nanoprinting. One example is the light cage^11,12^, which consists of a periodic arrangement of polymer strands surrounding a central hollow core^13^. This on-chip structure resembles the geometry of an all-solid bandgap fiber (e.g., ref. ^14^) and is used for various spectroscopic and bioanalytical applications^12,15,16^. Another example is the antiresonant microgap waveguide^17^, which is composed of square hollow-core segments separated by gaps that allow external access to the core region^18^. This structure exhibits improved light-guiding performance such as wide transmission bands and low losses, making it a promising approach for novel on-chip HCWs^18^.

Further photonic integration and greater handling flexibility can be achieved by interfacing HCWs with commercially available optical fibers, eliminating the need for free space components and expanding the range of potential applications. In this regard, we have recently demonstrated the interfacing of light cages with fibers^19^, while another recent study shows photonic crystal fiber-like elements on fiber end faces for applications such as mode conversion^20^. Taken together, these works demonstrate that the concept of interfacing HCWs with fibers is a promising strategy to significantly enhance photonic integration and pave the way for the development of novel sensing devices.

Nanoparticle Tracking Analysis (NTA) is a key technique in nanoscale science for characterizing nanoparticles (NPs) and revealing dynamic processes at the single particle level. Conceptually, NTA relies on tracking the Brownian motion of diffusing NPs and analyzing their trajectories. Since the NTA is based on a statistical analysis, it is necessary to record very long trajectories for a highly accurate determination of for instance the NP diameter^21^. This method is widely applicable in various fields, including bioanalytics^22,23^, life sciences^24^, environmental sciences^25^, and nanoscale materials science^26^.

Most NTA implementations use bulky setups where neither the excitation beam nor the NPs are confined, resulting in short trajectories and high statistical inaccuracy. Waveguide-based optofluidics can solve this problem, enabling low-cost, high-speed devices with small geometric footprints. HCWs are particularly suitable because they allow NP illumination via the core mode, resulting in homogeneous, constant, and well-defined illumination over the entire field of view, making them particularly promising for fiber and waveguide-based NTA (FaNTA).

A limited number of HCW-based FaNTA implementations have been documented, mainly in the field of fiber optics. Examples include the detection of bacteriophages in antiresonant hollow-core fibers^27^ or the characterization of mixed NP ensembles in single-anti-resonant element fibers^28^. In planar photonics, the number of studies is substantially smaller. One study characterized single NPs using on-chip ARROWs^29^, while another reports on NTA with locally structured light cages to measure solvent-induced shell collapse of functionalized NPs^16^. Note that in the latter study, the degree of fiber integration was limited due to a V-groove chip. These results clearly demonstrate the critical role of coupling HCWs with fiber, especially for applications in NTA.

**Fig. 1:**
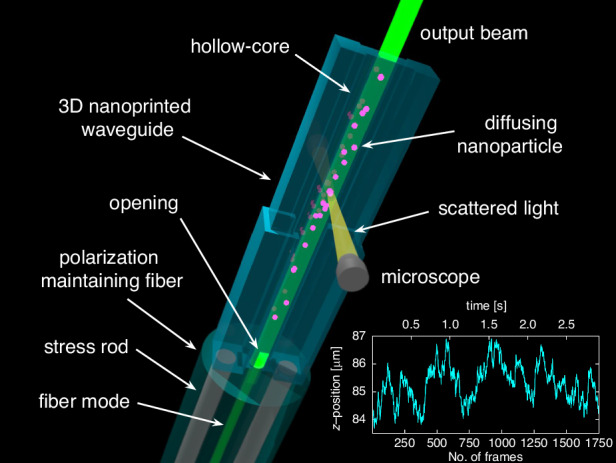
Illustration of the 3D nanoprinted square-core hollow-core waveguide (light blue) interfaced with a commercially available polarization-maintaining fiber (light green) and used for nanoparticle tracking analysis. The magenta elements indicate the nanoparticles that are diffusing inside the core of the waveguide and illuminated by the core mode (green). The yellow volume shows the light scattered by a nanoparticle and captured by the microscope (gray). The inset shows an example of a measured longitudinal trajectory of a selected gold nanosphere diffusing inside the waveguide core

Here, we introduce the concept of interfacing square-core HCWs with commercially available optical fibers using 3D nanoprinting (Fig. 1), and demonstrate for the first time the application relevance of such an integrated monolithic device in a nanoscience-related characterization technique, namely NTA (inset in Fig. 1). The study addresses all photonics-related aspects, including design strategy, experimental implementation details, elucidation of key principles (e.g., light guiding mechanism and mode excitation principle), optical characterization, and demonstration of application relevance.

## Results

### Waveguiding concept and waveguide-based nanoparticle tracking analysis

**Fig. 2:**
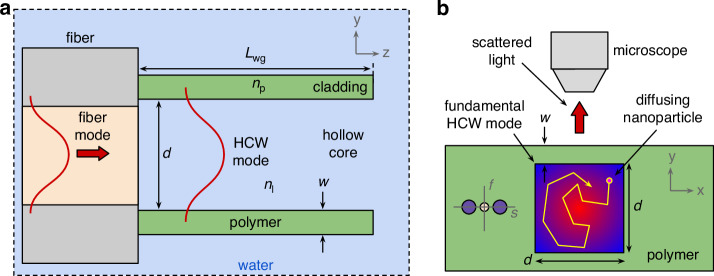
Schematic representation of the concept of fiber-interfaced square-core HCW. **a** Cross-section in the yz-plane (*x* = 0) with the different elements (light red: core, gray: cladding, green: polymer, light blue: water) and relevant parameters (*L*_wg_: length of waveguide, *d*: core size, *w*: strand thickness, *n*_p_: refractive index of polymer, *n*_l_: refractive index of liquid medium). The red lines show the fundamental mode in fiber and HCW. **b** Cross-section in the *xy*-plane within the HCW for the configuration used in the NTA experiment. The yellow line shows an example trajectory of the diffusing NP (yellow-magenta circle) within the core of the HCW. The red arrow indicates the light scattered by the NP and detected by the microscope. The sketch in the left part of the HCW refers to the orientation of the polarization maintaining fiber (f: fast axis, s: slow axis) relative to the HCW (purple disks: stress rods, light red disk: fiber core)

Within the *yz*-plane, the cross-section of the waveguide geometry used in this work (Fig. 2(a)) consists of two high-index layers (refractive index:*n*_*p*_, width *w*) that are separated by the distance *d*) and embedded in the liquid medium (refractive index: *n*_*l*_). The key to guiding light in such a HCW is the anti-resonance effect, which exploits the strong reflection of light from thin dielectric layers at near-grazing incidence^30^. This effect is based on interference, resulting in a sequence of transmission bands separated by resonances in the spectral transmission distribution. As shown in ref. ^31^, the spectral position of these resonances is determined by the modal anti-crossing of the core and layer modes and can be described by the following equation^17^:

1 $${\lambda }_{R}=\frac{2w}{m}\sqrt{{n}_{p}^{2}-{n}_{l}^{2}}$$

where *m* is the order of the layer mode. Equation 1 clearly shows that the resonance wavelength and the spectral intervals of high transmission can be very precisely adjusted by the thickness of the layer. Note that the confinement along the *x*-direction relies on single-interface reflection and is therefore not resonant.

The concept discussed here is based on the precise positioning of the HCW on the core of the fiber used. In relation to NTA, it is important to note that the power of the light scattered by the NPs strongly depends on the light polarization at the location of the particle. Therefore, a polarization-maintaining single-mode fiber (PM460-HP, PMF) was employed, with the cross-section of the HCW aligned with the optical axis of PMF (Fig. 2b). FaNTA involves recording the diffuse motion of NPs by imaging the laterally scattered light and statistically analyzing the trajectory using mean-square-displacement (MSD) analysis. Two points are crucial for the accurate determination of NP diameter: (i) The image acquisition must be as aberration-free as possible in order to minimize the localization error. (ii) The diffusion of the NP should be spatially confined to maximize observation time to minimize the high statistical error. This effect results from the Cramer-Rao lower bound, showing that the standard deviation of the determined hydrodynamic diameter mainly depends on the inverse the of number of available frames ${\sigma }_{d}\propto \sqrt{1/N}$, (*N*: number of frames (i.e., images) per trajectory)^28^.

The fiber-interfaced HCW concept discussed in this paper effectively addresses both issues. Aberration-free imaging is achieved by positioning the polymer membrane perpendicular to the microscope axis and by the absence of complex microstructural elements in the beam path. In addition, the rectangular cross-section of the HCW limits transverse diffusion, allowing for substantially longer observation times in contrast to open waveguide structures^16^.

### Implementation strategy and NTA experiments

**Fig. 3:**
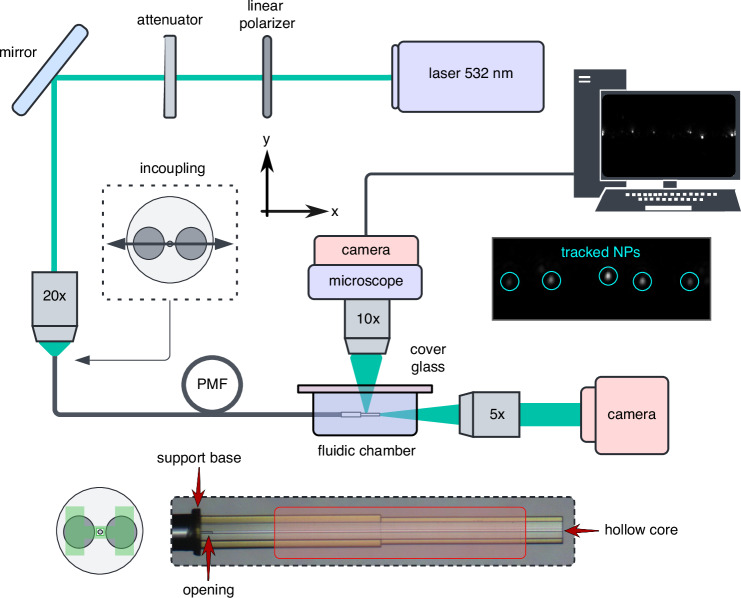
Optical setup used for the NTA experiments (detailed description in the main text). The right inset shows a selected image with five tracked nanoparticles (mean diameter: 100 nm). The left inset shows the orientation of the PMF with respect to the polarization of the input light (double arrow). The lower part of the figure shows (right) a microscopic side view of the fiber-interfaced HCW and (left) the orientation of the HCW relative to the PMF. The tracking experiments were performed in the red area

The HCWs were fabricated using 3D nanoprinting via direct laser writing, allowing for the creation of precise submicron features directly on the end faces of the PMF. The process was optimized to ensure high structural accuracy and effective resin removal (see Methods section for details). Finite element modeling (FEM) simulations were conducted using commercial software to analyze the intensity distribution and attenuation of various leaky modes within the HCW. These simulations provided key insights into mode polarization and coupling efficiency between the fundamental fiber and HCW modes (see Methods for more information). The optical characterization of the HCW involved the use of a broadband light source with polarization control, along with diagnostic tools (e.g., spectrum analyzer, camera) to analyze the transmitted light and mode profile in air and aqueous environments (see Methods for more information). To demonstrate the application potential of the fiber-interfaced HCW in NTA, two ensembles of ultra-uniform gold nanospheres with average metallic diameters of 50 nm and 100 nm were used as test objects in an aqueous solution. The nanoparticle concentration was carefully adjusted to ensure that approximately 25-50 particles were present within the HCW core observation volume (see Methods for further details). The NTA experiments used a setup consisting of a cw-laser, coupling optics, and a microscope for image acquisition (Fig. 3, c.f. Methods sec.). Polarized light (*λ*_0_ = 532 nm) was coupled into the PMF, with the HCW positioned in a fluidic chamber containing the NP solution. Proper launching conditions were ensured by measuring the mode profile at the HCW output. Imaging of the NP diffusion within the waveguide core was performed using transverse microscopy with a high-speed camera, capturing 400 and 600 frames per second for the 50 nm and 100 nm gold nanosphere ensembles and leading to tracking times of 20 and 10 seconds (8000 frames and 6000 frames). Note that the core was aligned perpendicular to the microscope axis, allowing a total length of 700 µm to be imaged along the *z*-axis (red area Fig. 3). As shown by sequential ray tracing simulations with Zemax that account for light propagation through the microscope assembly (for details see Sec. S8-A in the Supplementary Information), precise alignment is critical to achieving high image quality. If none of the optical components, in particular the coverslip between the specimen and the microscope objective, are tilted, the HCW system discussed here provides diffraction-limited image quality.

### Optical properties of the fiber-interfaced HCW

For the NTA experiments presented later, it is crucial to achieve a high purity of the excited mode to ensure that the longitudinal illumination of the NPs is as homogeneous as possible. This is achieved here by using a PMF that provides a Gaussian input mode with well-defined linear polarization. This arrangement results in the dominant excitation of the polarization-matched fundamental modes of the HCW. The effectiveness of this approach is quantified by the calculated coupling efficiency, which is >0.7 when the polarization-matched fundamental mode of the HCW is excited, while all other modes show significantly lower efficiencies (see Supplementary Information, Sec. S1). This high coupling efficiency results not only from the intensity distribution but also from the spatial phase distribution of the HCW mode. In particular, only the fundamental HCW-modes include a constant spatial phase distribution, similar to the phase distribution of the fiber mode^32^.

**Fig. 4:**
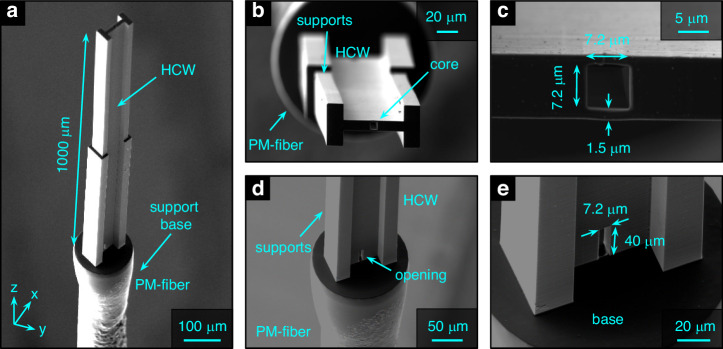
SEM images of an example of a square-core hollow-core waveguide nanoprinted on the end face of a PMF. **a** Oblique view of the entire HCW including PMF. **b** Top view. **c** Close-up view of the hollow core section, including the dimensions of the key elements. **d** Tilted top view of the opening at the bottom of the waveguide. **e** Close-up view of the opening, including dimensions

The HCW was printed on the end face of the mentioned PMF and optically characterized in both air and water-filled environments. The printed HCW has an overall length of *L*_*wg*_ = 1000 µm and is mechanically stabilized by solid two-stage blocks intersecting at roughly 500 µm (Fig. 4a and b). The hollow core has dimensions of 7.2 µm x 7.2 µm (Fig. 4b and c), with the polymer membranes having a thickness of approximately *w* ≈ 1.5 µm in the *y*-direction. On the bottom of the waveguide, openings were introduced on both sides of the core to allow access for the developer and to facilitate the flow of the nanoparticle solution within the waveguide (Fig. 4d and e).

**Fig. 5:**
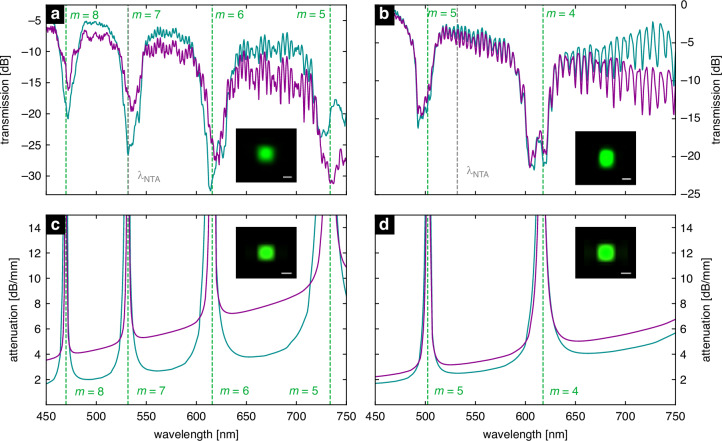
Measured and simulated spectral distribution of the power transmission of the fundamental core mode of the fiber-coupled HCW. Experimental transmission spectra of HCW when exposed to (**a**) air or (**b**) water. The vertical grey dashed line indicates the operating wavelength for the NTA experiments (*λ*_NTA_ = 532 nm). The insets show the corresponding spatial intensity distribution at *λ*_0_ = 525 nm (*x*-polarization). The bottom plots (**c**, **d**) show the simulated fundamental mode attenuation distributions in air and water, respectively, with insets showing the simulated mode at *λ*_0_ (scale bar is 4 μm). In all plots, the dark cyan and purple curves represent the fundamental modes being polarized with the electric field in *y*- or *x*-direction, respectively. The vertical green dashed lines indicate the resonance wavelength calculated by Eq. 1

All measured spectra show an alternating sequence of high and low transmission intervals (Fig. 5a and b), clearly indicating the antiresonance effect. Note that the measured spectra are normalized to the source spectrum for the corresponding polarization. This light guiding mechanism is further confirmed by the exact correspondence of the transmission minima with (i) the resonance positions calculated according to Eq. 1 and (ii) the maxima of the simulated attenuation of the fundamental mode (Fig. 5c and d). In addition, the intensity distribution of the measured mode agrees with the simulated fields of the fundamental mode, demonstrating that a selected mode can be excited with high purity, consistent with the simulations of the field distributions in the HCW. This is in good agreement with mode overlap simulations showing that the fundamental HCW modes are excited to a very high degree by the corresponding fundamental polarization-matched fiber mode (see Supplementary Information, Sec. S1). Modal losses were determined by fabricating waveguides of different lengths on planar glass substrates (waveguide axis perpendicular to the surface) and the measuring polarization-resolved power transmission (see Supplementary Information, Sec. S6). Losses were quantified by averaging power values within two spectral intervals, plotting them against length, and applying linear fitting. The slope represents the modal loss and was approximately 1–2 dB/(100 µm), which is reasonable for the device length used here. Note that these losses are about 10 times higher than other on-chip hollow-core waveguides (e.g., light cage: ~1 dB/mm^11^, ARROW: ~1 dB/mm^9^, which is due to reduced mode confinement along the x-direction caused by the lower reflectivity of the single-layer interface. The losses of modes with vertical polarization (y-direction) are generally lower, benefiting from the higher reflectivity of the membrane interface. It is interesting to compare the two refractive index environments: for water, the transmission bands are significantly broader, which is a consequence of the changed refractive index and the modified dispersion of the membrane modes. The very large bandwidth (in some cases >100 nm) in water is significantly larger than in the comparable light cage system, making this type of waveguide attractive for various spectroscopic applications^33^. Another difference in the modal loss for the two polarizations (the difference between the curves in a plot), where a water environment shows a smaller difference. This can be explained by a ray model^17,30^, which correlates the modal attenuation to the reflection of a single light ray at the membrane. As the refractive index contrast decreases, the magnitude of the reflection of the two polarizations becomes more similar, resulting in a smaller difference in modal attenuation.

### Application in nanoscience analysis

To demonstrate the practical relevance of the discussed waveguide system in nanoscale analysis, the fiber-interfaced HCW was used for NTA to characterize NP ensembles. Within NTA, the trajectory of each NP was obtained by image processing of the received videos and analyzed by mean-square-displacement (MSD) analysis (c.f. refs. ^16,34^). In detail, the *z*-position of the trajectories of each NP was analyzed using

2 $$\left\langle {z}^{2}\right\rangle =2{D}_{z}\Delta t$$

with the lag time *∆t*, the axial MSD $\left\langle {z}^{2}\right\rangle$, and the diffusion coefficient *D*_*z*_. Note that the symbol ${{\langle \; \rangle }}$ denotes the ensemble mean. The resulting diffusion coefficient is then converted into the hydrodynamic diameter via the Einstein-Stokes relation:

3 $${d}_{h}=\frac{{k}_{B}T}{3{\rm{\pi }}{\rm{\eta }}{D}_{z}}$$

where *k*_*B*_ is the Boltzmann constant, *T* is the absolute temperature, and η is the dynamic viscosity of the fluid. Note that the MSD value of a single lag-time represents a statistical average of position differences over the entire trajectory, not a single value. For example, the shortest lag-time ∆*t* averages *N*-1 MSD values, providing high statistical significance. Longer lag-times use fewer values, reducing significance and making the first few lag-times most relevant. Details of the MSD analysis can be found in the Supplementary Information of^35^. Here two lag times have been used due to the high signal-to-noise ratio the tracked NPs in the images and the fast diffusion^21^. The resulting NP diameter distribution is displayed as a histogram and key parameters (mean hydrodynamic diameter, inverse coefficient of variance) are determined. Note that the coefficient of variation (CV) is a measure of relative variability, calculated as the standard deviation divided by the mean. Statistical accuracy is improved by filtering short trajectories and using the z-score method to remove outliers (see Supplementary Information Sec. S3). Due to the presence of the polymeric membranes, all results are corrected for confined diffusion by taking into account the hindrance factor (c.f. Supplementary Information Sec. S2).

**Fig. 6:**
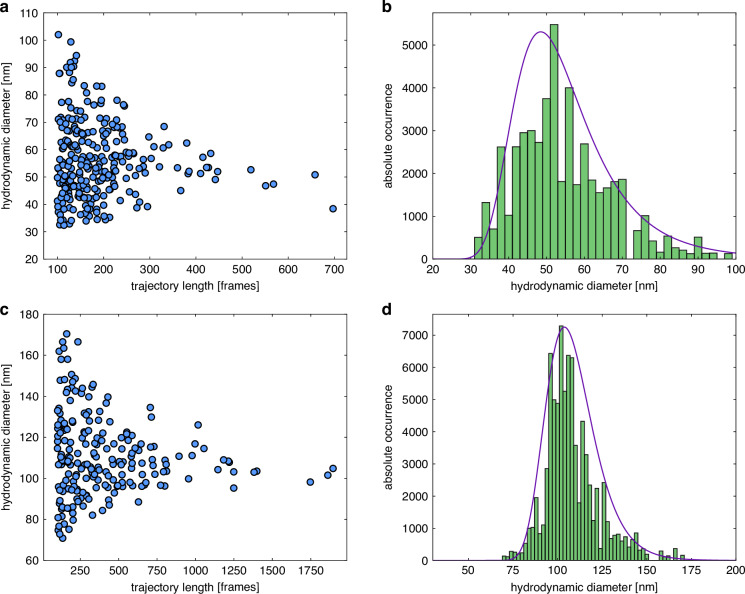
Results of NTA experiments with the fiber-interfaced HCW for two ensembles of gold NPs. The top row refers to NPs (ensemble 1) with an average diameter of 50 nm NPs, the bottom to NPs (ensemble 2) with an average diameter of 100 nm. The left column shows the distribution of the determined hydrodynamic diameter as a function of the track length for (**a**) 50 nm NPs and (**c**) 100 nm NPs. The right column shows the corresponding weighted (by track length) histograms (green bars) used to extract the main parameters including inverse Gaussian fits (purple lines) for the (**b**) 50 nm NPs and (**d**) 100 nm NPs. Note that the absolute occurrence (*y*-axis) represents the probability multiplied by the trajectory length, which statistically results in longer trajectories being more relevant

Specifically, the device was applied to the two different types of gold NP solutions, yielding more than 100 different trajectories, each of which was analyzed using MSD-based NTA to obtain the corresponding hydrodynamic diameter (Fig. 6a and c). The resulting nose-like distributions are characteristic of NTA experiments and indicate the acquisition of very long trajectories (*N* > 300), leading to high statistical significance in the determined hydrodynamic diameters. The corresponding histograms (Fig. 6b and d) show an inverse Gaussian evolution (represented by the purple curves) and provide essential parameters for the investigated NP ensembles (Table 1).

**Table 1:** Summary of the key parameters of the NP ensembles studied, determined by MSD-based NTA using the fiber-interfaced HCW discussed here

	metallic diameter (manufacturer, TEM)	mean hydrodynamic diameter	CV	mean hydrodynamic diameter (dilution)	CV
method	TEM	HCW-NTA	HCW-NTA	DLS	DLS
ensemble 1	49.8 nm	55.5 nm	0.22	59.9 nm	0.23
ensemble 2	102.2 nm	109.0 nm	0.13	112.0 nm	0.24

For comparison, the corresponding values measured with a commercial device using dynamic light scattering (DLS) are additionally presented

The values for the mean ensemble diameter obtained for the HCW-based NTA are realistic because they are slightly higher than the metallic diameter (*d*_*m*_ < *d*_*h*_) due to the formation of the electric double layer around the metallic core of the NPs in the liquid environment and the covalently bonded PEG12-COOH ligands (PEG length 5.5 nm), which allow functionalization with e.g. biomolecules. The measured mean diameters are in good agreement with those from the DLS reference measurements and experiments with a commercially available NTA-device (NanoSight NS300), clearly demonstrating the applicability of the fiber-interfaced HCW in nanoscale analysis (a detailed comparison between the HCW-NTA approach and the NS300 can be found in the Supplementary Information, Section S10). It should be noted that DLS measurements must be performed at much higher NP concentrations, which naturally results in different NTA and DLS diameters. This seems to be particularly evident for the 50 nm NP solution, which was concentrated to obtain a measurable signal in the DLS experiments. Particularly noteworthy is the high accuracy of the NTA measurements, as reflected by the lower CV compared to the DLS reference, especially in the case of the 100 nm NP ensemble. Note that compared to DLS, NTA minimizes the effects of particle-particle interactions, avoids intensity weighting and thus unwanted favoring of larger particles, provides less biased size distributions by detecting individual particles, is capable of handling sample polydispersity and provides measurements at biologically relevant concentrations (details can be found in Sec. S5 of the Supplementary Information).

Diffraction-limited imaging quality of the nanoparticles, with a non-saturated and fully circular light distribution (inset in Fig. 3), was achieved, slightly exceeding the quality observed in images of a reference measurement conducted with a commercially available NTA instrument (NanoSight NS300, for details, see Sec. S8-B) of the Supplementary Information).

## Discussion

It is important to note that the performance of the present NTA scheme crucially depends on the nearly aberration-free imaging of the NPs within the core of the HCW (example of a selected frame is shown in the inset of Fig. 3a). This is due to the unique properties of the HCW including non-curved interfaces, the perpendicular orientation of the polymer membrane to the microscope axis, and the absence of complex microstructured elements in the microscopic beam path (for details see the ray-tracing simulations in Sec. S8-A of the Supplementary Information). Together, these factors ensure high-quality imaging by minimizing the distortions and aberrations typically encountered in other configurations. Because of these properties, our approach is principally able to detect nanoscale species with very high temporal resolution, as for instance demonstrated in the work of Špačková et al^36^. That NTA-related study introduces nanofluidic scattering microscopy, a label-free technique for real-time imaging and molecular characterization of freely diffusing biomolecules and extracellular vesicles within nanofluidic channels (a comparison of the key features of both approaches can be found in the Sec. S8-C of the Supplementary Information). The discussed HCW approach can be valuable in this context, as the local structuring of the HCW – achieved by adjusting the nanoprinting strategy – allows the generation of customized light fields with properties tailored to the mentioned applications.

The track lengths achieved in our work are comparable to those reported in other NTA-related studies (see Sec. S9 of the Supplementary Information) including the work of Špačková et al.^36^, with our system operating at a significant higher frame rate of 400 fps compared to 25-30 fps (Table S5), and 200fps in the work of Špačková et al. (Table S4).

The fiber-interfaced HCW concept demonstrates a high degree of integration and suggests potential applications of the discussed approach in various fields such as bioanalytics (e.g., on-chip analysis^37^), environmental science (e.g. gas sensing^38^) quantum technologies (e.g., light/matter interaction in alkali vapors^39,40^), optical manipulation (e.g. particle transport^41^) and life sciences (e.g., pharmaceutical monitoring^7^). From an optofluidic perspective, this approach paves the way for novel all-fiber devices with enhanced functionality for microfluidics^42^. In addition, applications in the context of gas-based photonics can be anticipated, exploiting the enhanced light-matter interaction and advanced waveguiding properties of hollow-core waveguides^43^. The small overlap between the optical field and the polymer makes these waveguides ideal for mid-IR applications, such as fingerprint spectroscopy in liquid and gaseous environments^44^, and Raman spectroscopy^45^.

The sensing capabilities of the HCW can be enhanced by increasing its length, which requires reducing the confinement loss along the single interface direction (*y*-direction). This can probably be achieved by incorporating additional microstructured anti-resonant elements into the waveguide system, which is the subject of current investigations. Furthermore, the functionalization of the waveguide with active molecules can extend its application potential towards fields such as photochemistry^46^ and plasmonics^47^.

Integration can be greatly improved by adding a reflective mirror at the end of the HCW (by printing an additional element and low temperature gold deposition), allowing the probe light to be transmitted and received through the same PMF, leading to full fiber integration via a fiber circulator that can be important for applications beyond NTA, such as integrated liquid or gas spectroscopy detection^15^ or alkaline vapor based reference cells^12^. In addition, by connecting both ends of the HCW to fibers using a ferrule-type arrangement, any free space components are eliminated, resulting in an all-fiber device.

As demonstrated in our previous work^32^, highly efficient selective mode excitation inside the HCW can be achieved by introducing phase plates at the waveguide input. This method can be applied to the discussed device by nanoprinting phase elements, complex phase-only holograms^48^, or even metasurfaces^49^ on the fiber core section expanding the range of potential applications within for instance in quantum optics^39^ and life sciences^37^.

In summary, we have successfully introduced the concept of interfacing square-core HCWs with commercially available optical fibers using 3D nanoprinting and demonstrated its practical relevance through a nanoscience characterization technique. This innovative integrated photonics approach results in a monolithic, fully fiber-integrated device with key advantages, including alignment-free operation, high-purity fundamental mode excitation, full polarization control, and exceptional handling flexibility. For the first time, the potential of a fiber-interfaced waveguide in nanoscale analysis has been demonstrated through NTA experiments. The fiber-interfaced HCW concept holds great promise for applications in bioanalysis, environmental sciences, quantum technologies, optical manipulation and life sciences. In addition, it paves the way for the development of novel all-fiber devices that exploit enhanced light-matter interactions, providing a compact and flexible solution suitable for remote operation.

## Materials and Methods

### Fabrication of waveguides/nanoprinting

The HCWs were fabricated by 3D nanoprinting using direct laser writing with a commercial nanoprinter (GT2, Nanoscribe GmbH) and the photoresist IP-DIP2, which was chosen for its ability to realize submicron features. The waveguides were written vertically on the PMF endfaces (z-direction in Fig. 2) with a 50 nm hatching distance and 100 nm slicing distance, taking approximately 10 hours. To ensure proper resin removal, the printed structure was immersed in propylene glycol methyl ether acetate (PGMEA) for 30 minutes, repeated with fresh PGMEA, and then immersed in Novec for 2 minutes.

### Simulations

Finite element modeling (FEM) simulations were performed using commercial solvers (COMSOL Multiphysics and JCMwave) to analyze the intensity distribution and attenuation of different leaky modes inside the HCW (*d* = 8 μm, *w* = 1.56 µm, refractive indices of water and polymer obtained from^15^). The simulations computed the eigenstates within the *xy*-plane (simulation volume 30 μm × 30 μm), using perfectly matched layer (PML) boundary conditions to absorb transverse light (minimum mesh size 45 nm). The resulting modes were labeled based on polarization and effective index^50^. The coupling efficiency between the fundamental fiber mode and HCW modes was determined by calculating the overlap integral of the relevant modes^51^. The simulations assumed a core diameter of 3 µm, with core and cladding refractive indices (at *λ*_0_ = 532 nm) of 1.4656 and 1.4607, respectively, resulting from the numerical aperture (NA = 0.12) of the PM-460HP fiber.

### Optical Setup (for optical characterization)

The optical characterization of the HCW was performed using a broadband light source, polarization control, the sample, and diagnostics. In detail, polarization-controlled light from a supercontinuum source (SuperK COMPACT, NKT Photonics) was coupled into the PMF (PM460-HP) with a 20x aspherical lens (NA 0.5). The transmitted light from the HCW was collected with a 10x aspherical lens (NA 0.3) and analyzed with an optical spectrum analyzer (AQ-6315A, Ando). The mode profile was recorded with a CCD camera using a 525 nm bandpass filter. When necessary, the HCW and part of the PMF were immersed in water to create an aqueous environment.

### Nanoparticle solution

To demonstrate the application potential of fiber-interfaced HCW in the context of NTA, two ensembles of ultra uniform gold nanospheres (nanoComposix) with average metallic (diameter of the metal volume, determined by TEM of manufacturer) diameters of ${d}_{m}^{1}=49.8\pm 1.8$ nm (referred as 50 nm NPs) and ${d}_{m}^{2}=102.2\pm 4.2$ nm (referred as 100 nm NPs) were used as test objects in an aqueous solution. To prevent aggregation of NPs, the particles are placed in a 2 mM sodium citrate water solution. The dilution factor is chosen so that there are approximately 25-50 NPs in the observation volume defined by the core of the HCW.

### Optical setup (for NTA experiments)

The NTA experiments include a setup that consists of a cw-laser, coupling optics, and a microscope for image acquisition (Fig. 3). Specifically, light from a laser (WhisperIT W532-50FSl (Pavilion Integration Corp.), wavelength *λ*_*l*_=532 nm) was polarized along the x-axis and coupled into the PMF (PM460-HP) with an aspherical lens (20x, NA = 0.4). The HCW at the other end of the PMF was placed in a homemade fluidic chamber containing the NP solution, enabling liquid solution filling via capillary forces^52^, verified by monitoring the core mode via a camera and lateral microscopy (for details on filling and replacing fluids, see Supplementary Information Sec. S7). To ensure proper launch conditions, the mode profile at the output of the HCW was additionally measured. Transverse imaging of NP diffusion inside the waveguide core was performed microscopically with a 10x objective (10x, NA = 0.25, focal length of 18 mm, depth of focus of 440 µm) connected to a fast camera (Basler acA4096-40um, frame rate = 400 & 600 frames per second, exposure time = 0.2 ms & 1 ms for the 50 nm and 100 nm gold nanospheres). The core of the waveguide was centered and aligned in the field of view of the microscope, allowing a total length of 700 µm to be imaged in the z-axis direction. The tracking time for the 50 nm and 100 nm NPs was 20 seconds (8000 frames) and 10 seconds (6000 frames), respectively. The spatial domain in which the tracking has been performed is highlighted by the red area in the microscopic side view of the HW shown in the bottom part of Fig. 3.

## Supplementary information

Supplementary Information for “3D Nanoprinted Fiber-Interfaced Hollow-Core Waveguides for High-Accuracy Nanoparticle Tracking Analysis'

## Data Availability

The data supporting the results of this study are available from the corresponding authors upon reasonable request.

## Code availability

The code used in this study is available from the corresponding authors upon reasonable request.
