# Supplementary material for: 3D nanoprinted fiber-interfaced hollow-core waveguides for high-accuracy nanoparticle tracking analysis
Source: Light Sci Appl. 2025 May 15;14:197. doi: 10.1038/s41377-025-01827-9 (PMC12081878; doi:10.1038/s41377-025-01827-9)
Supplement: Supplementary file 1 — Supplementary Information for “3D Nanoprinted Fiber-Interfaced Hollow-Core Waveguides for High-Accuracy Nanoparticle Tracking Analysis' [file 41377_2025_1827_MOESM1_ESM.docx]

**Supplementary Information for “*3D Nanoprinted Fiber-Interfaced Hollow-Core Waveguides for High-Accuracy Nanoparticle Tracking Analysis*”**

*Authors*

Diana Pereira¹², Torsten Wieduwilt¹, Walter Hauswald¹, Matthias Zeisberger¹, Marta S. Ferreira², Markus A. Schmidt¹³⁴*

*Affiliations*

^1^Leibniz Institute of Photonic Technology, Albert-Einstein-Str. 9, 07745 Jena, Germany

^2^i3N & Physics Department, University of Aveiro, Campus de Santiago, 3810-193 Aveiro, Portugal

^3^Abbe Center of Photonics and Faculty of Physics, Friedrich-Schiller-University Jena, Max-Wien-Platz 1, 07743 Jena, Germany

^4^Otto Schott Institute of Materials Research (OSIM), Friedrich Schiller University Jena, Fraunhoferstr. 6, 07743 Jena, Germany

*Correspondence: Markus A. Schmidt. Email: [markus-alexander.schmidt@uni-jena.de](mailto:markus-alexander.schmidt@uni-jena.de)

Sec. 1: Key performance parameters of selected HCW modes

Table S1 shows the key performance parameters of the modes of the HCW at $\lambda_{0}$ = 532 nm simulated using FEM modeling (the intensity distributions of the $x$-polarized HCW and fiber (most right-handed plot) modes are shown in Fig. S1). The modes are ordered by the magnitude of their effective index and their dominant polarization direction along the $x$- or $y$-axis (coordinate system in Fig. 2). It is evident that higher-order modes show larger losses at a level of a few dB/mm. The $E_{11}^{y}$ mode shows the lowest losses, which is expected since this mode has minimal overlap with the single interface boundary along the $x$-direction and is confined by the polymeric membrane along the $y$-direction.

**
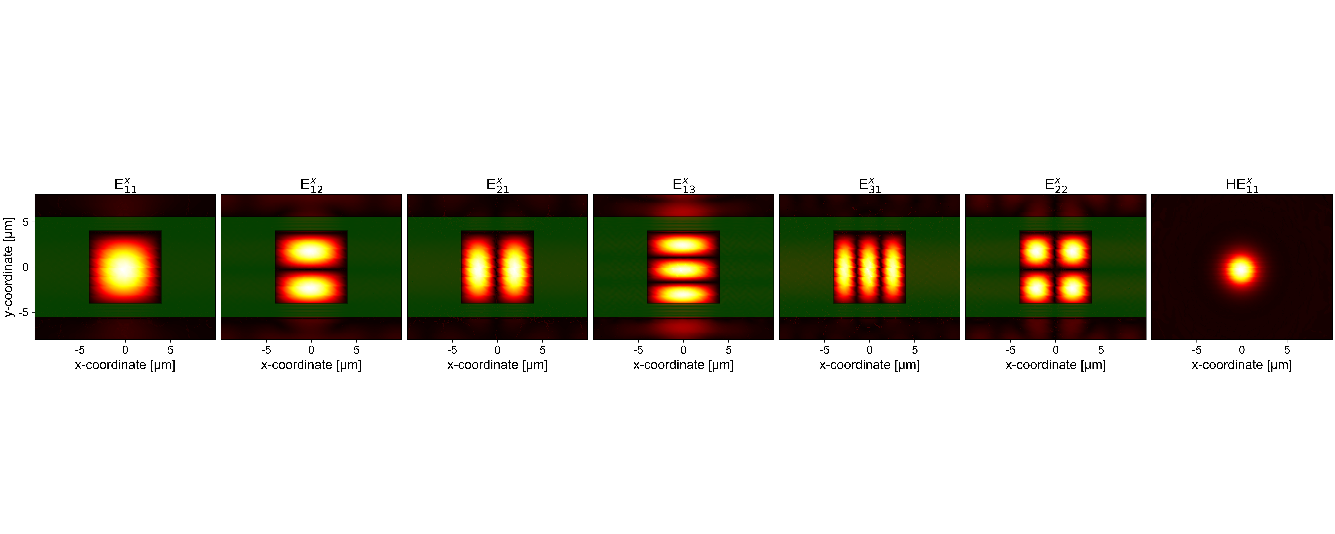
**

**Fig. S1:** Simulated spatial distribution of intensity of selected HCW modes at $\lambda_{0}$ = 532 nm. The color scale ranges linearly from zero (dark) to unity (light yellow). All plots are normalized to their respective maximum, i.e. the maximum value of each plot is one. The semi-transparent light green areas indicate the polymeric material. The distribution on the right refers to the $x$-polarized fundamental mode of the fiber.

Another important aspect is the coupling efficiency between the two linearly polarized fundamental modes of the fiber and the different HCW modes. The coupling efficiency is determined here by calculating the overlap integral of the fiber mode with the corresponding HCW mode^1^. The integration is limited to an area of 5 µm x 5 µm, which includes the majority of electromagnetic power, and the polarization of the modes is taken into account.

**Tab. S1:** Overview of the key performance parameter of various modes of the HCW at $\lambda_{0}$ = 532 nm (located in water), including mode type, polarization, real part of the effective refractive index (i.e. phase index), modal attenuation and coupling efficiency to the fundamental fiber modes (FM: fundamental mode, HOM: higher-order mode).

| Mode Name | Mode Type | Re(neff) | Attenuation [dB/mm] | Coupling efficiency (to HE_11_(x) fiber mode) | Coupling efficiency (to HE_11_(y) fiber mode) |
| --- | --- | --- | --- | --- | --- |
| $E_{11}^{x}$ | FM | 1.334668 | 3.122558 | 0.773 | 0.000 |
| $E_{11}^{y}$ | FM | 1.334670 | 2.315250 | 0.000 | 0.773 |
| $E_{12}^{x}$ | HOM | 1.333469 | 3.768533 | 0.000 | 0.000 |
| $E_{12}^{y}$ | HOM | 1.333511 | 3.607891 | 0.000 | 0.000 |
| $E_{21}^{x}$ | HOM | 1.333427 | 12.293508 | 0.000 | 0.000 |
| $E_{21}^{y}$ | HOM | 1.333436 | 9.366609 | 0.000 | 0.000 |
| $E_{31}^{x}$ | HOM | 1.331359 | 27.639165 | 0.0872 | 0.000 |
| $E_{31}^{y}$ | HOM | 1.331360 | 20.485240 | 0.000 | 0.0855 |
| $E_{13}^{x}$ | HOM | 1.331532 | 7.812198 | 0.0981 | 0.000 |
| $E_{13}^{y}$ | HOM | 1.331522 | 6.673426 | 0.000 | 0.0998 |
| $E_{22}^{x}$ | HOM | 1.332237 | 12.902282 | 0.000 | 0.000 |
| $E_{22}^{y}$ | HOM | 1.332249 | 11.736533 | 0.000 | 0.000 |

Sec. S2: Confinement on NP diffusion

An essential factor for accurately determining the hydrodynamic diameter of NPs is the effect of confinement on NP diffusion. This effect occurs because the viscosity of the fluid is not constant as the NP approaches the channel wall due to boundary effects. As a result, the viscosity increases near the channel wall, which can affect diffusion, especially in cases of small channels or large NPs. In cylindrical channels, the critical parameter is the ratio of the NP diameter to the channel diameter ($d_{p}$/$d_{c}$, where $d_{p}$ is the NP diameter and $d_{c}$ is the channel diameter). More details on the confinement effect can be found in ^2^. In this study, we account for this effect by using a local hindrance factor $K_{d}$ (less than one), resulting in a corrected diffusion coefficient $D_{corr}=D_{z}/K_{d}$. To calculate the hindrance factor in the HCW, we approximated the square-shaped cross-section of the core by a circle of radius 4 µm, which gives reasonable values for the hindrance factor. As shown in Tab. S2, the hindrance factor is 0.967 for 50 nm NPs and 0.942 for 100 nm NPs, indicating a negligible and moderate effect on diffusion for the NP ensembles considered.

**Tab. S2:** Summary of the hindrance factor and its influence on various key parameters for both NP ensembles.

| NP diameter | Local hindrance ($K_{d}$) | Hindrance corrected | Not corrected |
| --- | --- | --- | --- |
| 50 nm | 0.96663 | $\overline{d_{h}}$ = 55.5 nm | $\overline{d_{h}}$ = 57.4 nm |
|  |  | CV = 0.222 | CV = 0.226 |
| 100 nm | 0.94228 | $\overline{d_{h}}$ = 109.0 nm | $\overline{d_{h}}$ = 116.2 nm |
|  |  | CV = 0.130 | CV = 0.136 |

Sec. S3: Data processing using z-score

To improve the statistical relevance of the obtained results in our study, the z-score procedure is applied to the distribution of determined hydrodynamic diameters. The z-score is a statistical measure that indicates how many standard deviations an element is from the mean of the data set. Generally, the formula for calculating the z-score of a data point $x_{i}$ is:

$$\begin{aligned} z_{i}=\frac{\left( x_{i}-\mu\right)}{\sigma}\#\left( S1 \right) \end{aligned}$$

where: $x_{i}$ is the value of the data point, $\mu$ is the mean of the data set, and $\sigma$ is the standard deviation of the data set. The benefits of using z-score include:

- improved accuracy, as outliers are removed ensuring that the calculated mean and standard deviation are not biased by extreme values,
- improved reliability, as the refined data set provides a more reliable representation of nanoparticle diameters, resulting in better statistical significance in the analysis, and
- better consistency, as the z-score method provides a standardized way to identify and handle outliers, ensuring consistency in data processing steps.

The procedure used here in the context of MSD analysis addressed in this study includes the following steps

1. Calculation of the MSD: The MSD is calculated for each nanoparticle by tracking its Brownian motion over time.
2. Determination of the measured diameter distributions: The hydrodynamic diameter of each gold nanosphere is determined from the MSD analysis and using the Stokes-Einstein relation. This provides a distribution of measured diameters for the ensemble of nanoparticles under investigation.
3. Filtering by z-scores: To ensure the reliability and relevance of the measured diameter distribution, a z-score procedure is used, which includes
   1. Step 1: Calculation of the mean and standard deviation of the measured diameter distribution.
   2. Step 2: Calculation of the z-score for each measured diameter.
   3. Step 3: Identification and removal of outliers based on their z-scores. Typically, data points with z-scores greater than 3 or less than -3 are considered as outliers (i.e., they are more than three standard deviations away from the mean).

In this work, we removed the data points with z-scores > 2.576, resulting in the distributions that are shown in Fig. S2. Note that the resulting data sets are highly refined, as the removal of outliers using the z-score procedure results in more accurate and statistically relevant distributions of nanoparticle diameters.


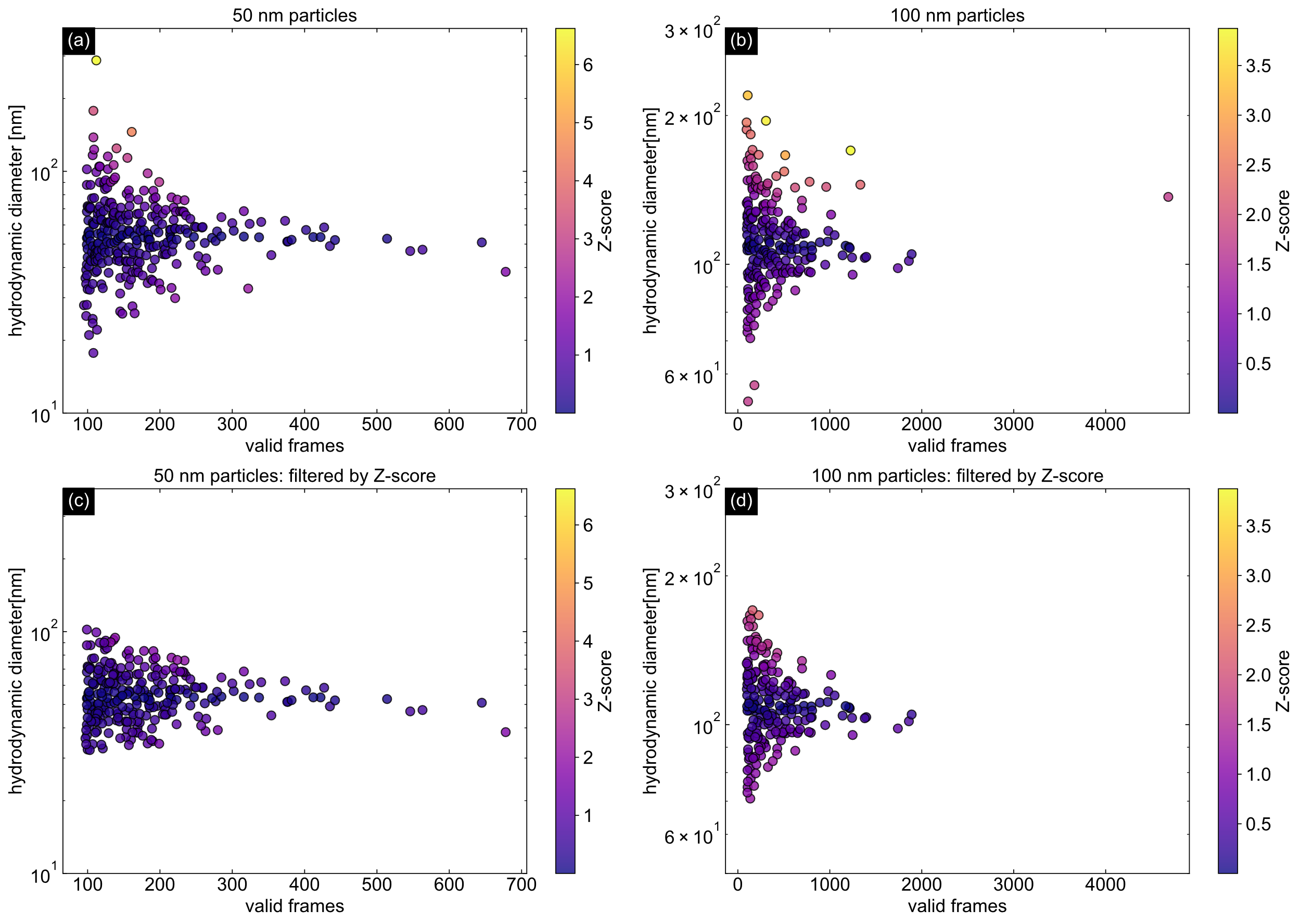


**Fig. S2:** Representation of the z-score filtering methods used here to increase the significance of the statistical data of the hydrodynamic diameter distributions of the investigated nanoparticle ensembles. The top row ((a) and (b)) shows the distributions directly after the MSD analysis before filtering, the bottom row ((c) and (d)) shows the distribution after applying the z-score method (left): Ensemble 1 (50 nm gold NPs), (a) and (c)), right: Ensemble 2 (100 nm gold NPs), (b) and (d))

Sec. S4: DLS Measurement

DLS measurements were performed using the ZetaSizer Nano-ZS (Malvern Instruments). Samples were loaded into 100 µl disposable microcuvettes and measured at 20°C. To achieve the manufacturer's recommended NP concentration (100 µg/ml), the diluted samples were first concentrated by centrifugation. Since the particle solutions were specified as "ultra uniform" (indicating a monodisperse sample with a Gaussian distribution), the cumulative analysis approach was used to determine the mean hydrodynamic diameter. This analysis provided the z-average diameter (intensity mean) and the polydispersity index (PDI). Assuming a Gaussian distribution, the relationship PDI = ($\sigma_{d_{h}}/\overline{d_{h}}$)² was used, allowing the coefficient of variation (CV) to be represented by the square root of the PDI. Note that the CV is generally defined as the ratio of the standard deviation to the mean and represents the relative variability. To increase the accuracy of the measurements, ten sets of measurements were made, each consisting of 18 individual runs.

Sec. S5: Comparison of DLS and NTA for nanoparticle characterization

When employing nanoparticle characterization techniques such as DLS and NTA, several critical factors influence the accuracy and reliability of the results. Among these, the following are particularly noteworthy:

- **Particle-particle interactions**: At the higher NP concentrations required for DLS, interparticle interactions such as agglomeration or aggregation can occur, leading to an apparent increase in the measured particle size. These interactions are less significant in NTA, where measurements are made at lower NP concentrations, minimizing such effects.
- **Intensity weighting:** DLS measures size based on intensity fluctuations in the scattered light, disproportionately weighting larger particles due to their greater scattering intensity. This is particularly important in the case of agglomeration, which can falsely dominate DLS measurements and lead to inconsistent results. NTA detects individual particles, providing a size distribution that is less biased by the presence of a few larger particles.
- **Sample polydispersity:** DLS averages the properties of the entire particle population, which can overestimate size if a polydisperse sample is present. NTA provides a more detailed size distribution by tracking individual particles.
- **Relevance to experimental conditions**: High NP concentrations in DLS may not represent biologically relevant or application-specific conditions, whereas NTA measurements at lower concentrations often better reflect realistic scenarios.

Sec. S6: Characterization of waveguide’s modal loss

To evaluate the modal losses in the HCW, transmission measurements were performed on waveguides fabricated on planar glass substrates, with the waveguide axis oriented perpendicular to the substrate surface. Identical waveguides of three different lengths were nanoprinted (Fig.S3), and polarization-resolved power transmission was measured (Figs.S4(a) and (b)).


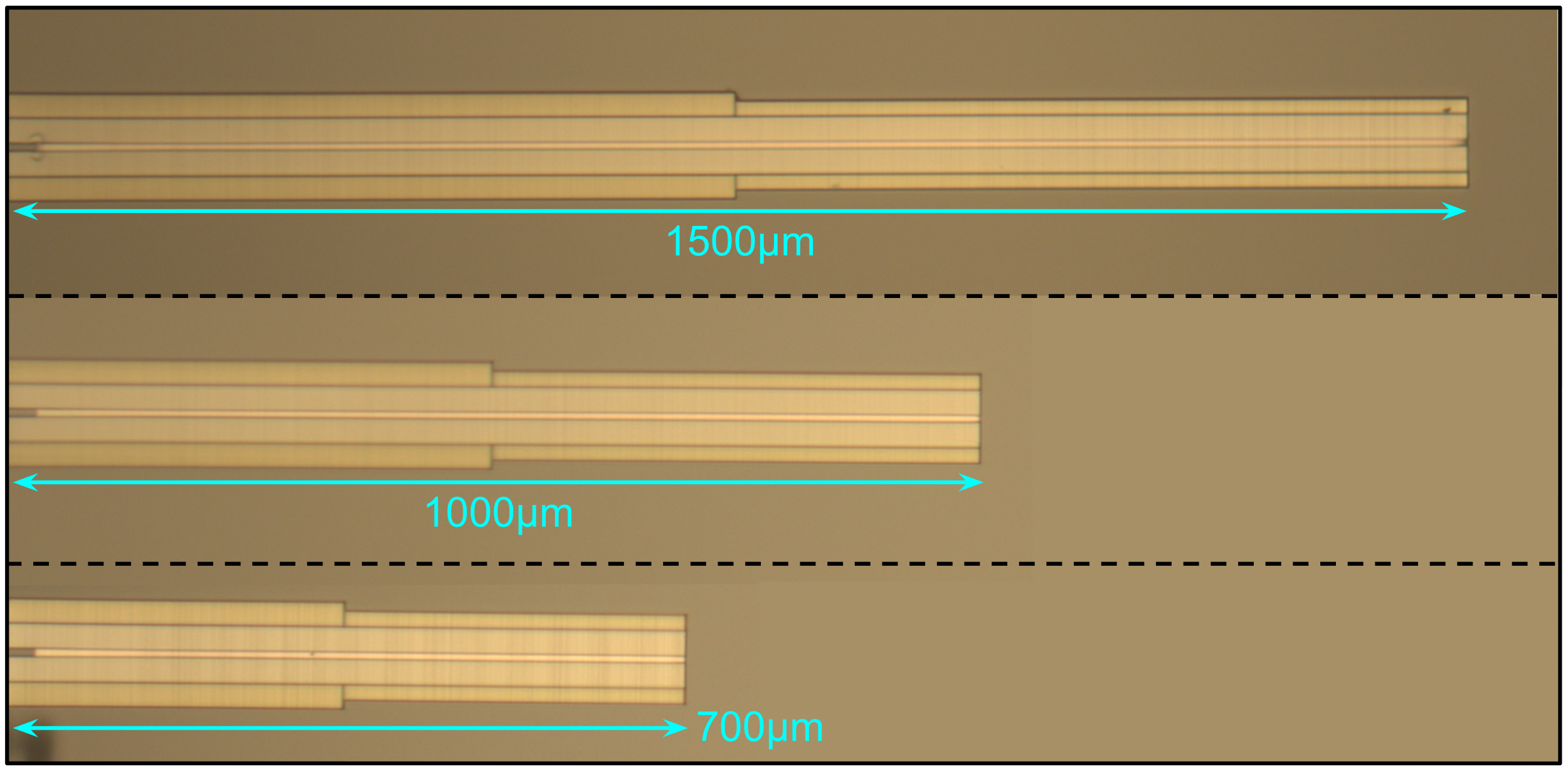


**Fig. S3:** Microscopic side view of three fabricated HCWs of different lengths on a planar substrate used to measure the spectral distribution of the attenuation of the fundamental mode (top: 1500 µm, middle: 1000 µm, bottom: 700 µm).

As expected, shorter waveguides showed higher transmission values. To quantify the losses, we (i) averaged power values within two spectral intervals located inside each transmission band to minimize the impact of the oscillations, (ii) plotted these averaged values (in dBm scale) against waveguide length, and (iii) fitted the data with a linear function. The slope of these fits provided the modal loss in dB/mm (Figs. S4(c) and (d)). Note that this approach is consistent with the standard cut-back method commonly used in waveguide and fiber loss characterization. The resulting losses (Tab. S3) are in the range of 10-20 dB/mm, which is higher than losses reported for other on-chip hollow-core waveguides (e.g., light cage: ~1 dB/mm, ARROW: ~1 dB/mm), primarily resulting from the reduced mode confinement along the x-direction caused by the lower reflectivity of the single-layer interface. Losses in the vertical direction (through the membrane) are comparatively smaller due to the higher reflectivity of the membrane-based interface. Note that the slight variations in membrane thickness across the three waveguides resulted in slight spectral shifts of the resonance dips in the spectral power distributions (Fig. S4(a) and (b)).


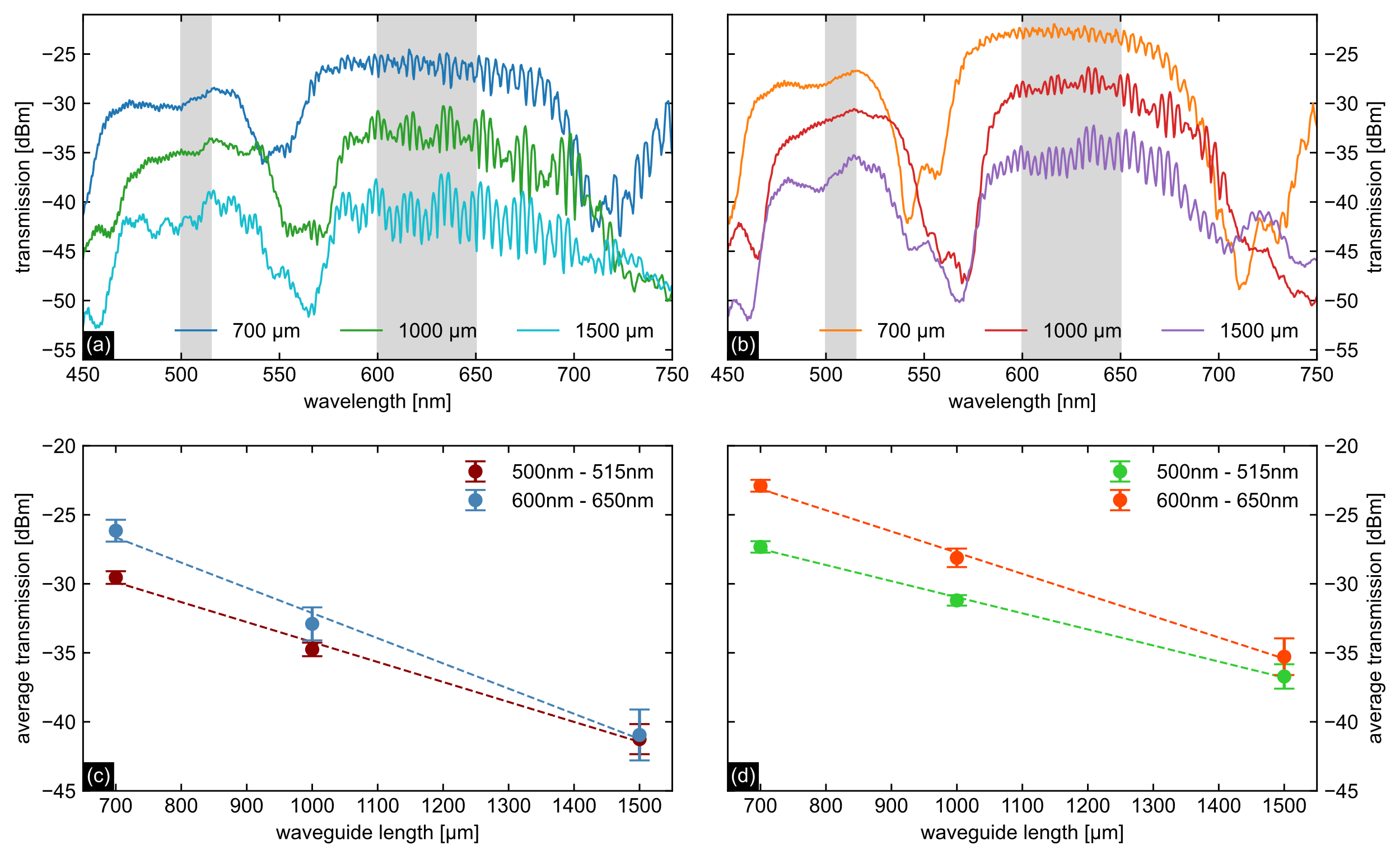


**Fig. S4:** Characterization of the modal attenuation (left column: horizontal polarization (hp, x-pol. In Fig.2(b)); right column: vertical polarization (vp, y-pol)). The top row shows the spectral distribution of the measured power transmission modal attenuation ((a): hp (x-pol). (b): vp (y-pol.)). The length of the respective waveguide is indicated by the legends. The bottom row shows the linear fits to the measured data points in two selected spectral intervals (shown in the respective top right corners), indicated by the gray areas ((c): hp (x-pol.), (d): vp (y-pol.)).

**Tab. S3:** Results of loss measurements on samples of various lengths (hp: horizontal polarization (x-pol.); vp: vertical polarization (y-pol.)).

|  | spectral interval [nm] | losses (vp)  [dB/mm] | losses (hp)  [dB/mm] |
| --- | --- | --- | --- |
| short wavelength band | 500 … 515 | 11.7 | 14.5 |
| long wavelength band | 600 … 650 | 15.4 | 18.3 |

Sec. S7: Handling of nanoparticle solutions in the HCW

1. **Insertion of liquid solutions into waveguide core**

The fiber, along with the nanoprinted structure at the fiber tip, was inserted into a custom microfluidic chamber, creating a closed environment for the liquid solution (an image of the experimental arrangement is shown in Fig. S5). The aqueous solution containing the nanoparticles was pipetted into the chamber, allowing it to enter the waveguide core by capillary forces, with a waiting time of approximately 2 minutes to ensure complete filling and stabilization of the experimental arrangement. Complete filling of the core section to reach a temporally stable situation was verified by (i) monitoring the core mode with the camera at the end of the waveguide during and after filling, and (ii) performing lateral microscopy to confirm filling and the presence of diffusing nanoparticles inside the core. Note that evaporation of the liquid was prevented by covering the top of the chamber with a glass cover. The chamber was refilled with solution as needed. Inserting a new fiber into this experimental setup was straightforward because the homemade microfluidic chamber was designed to fit on the ferrule where the fiber and sample were located, allowing easy integration of a new sample. To align the birefringence axis of the PANDA fiber with respect to the input laser polarization, the fiber input was adjusted using a rotatable mount and a microscope, while the coupling was optimized by measuring the output power with a power meter.


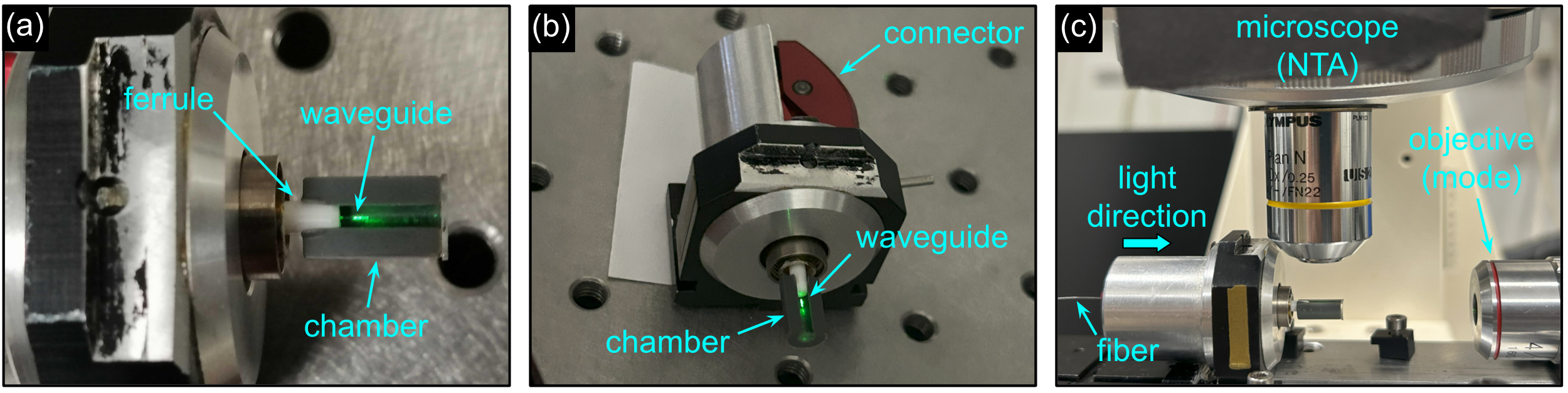


**Fig. S5**: Different views of the experimental setup used for the nanoparticle tracking experiments. (a) Top view of the chamber section including ferrule and fiber sample with the waveguide at the tip. (b) Corresponding angled view. (c) Arrangement for the nanoparticle tracking experiments, including the top microscope to investigate the nanoparticle diffusion inside the core and an objective to monitor the core mode.

1. **Replacement of liquid sample**

To introduce a completely new solution (e.g., for measuring a different particle size) into the waveguide core, the current solution was removed and the chamber was rinsed with isopropanol to clean the waveguide core section and the entire chamber. Liquid evaporation ensured complete removal of liquid and particles, resulting in a clean sample after one day, with no residual nanoparticles or dust observed in subsequent nanoparticle tracking experiments.

Sec. S8: Evaluation of aberration-free imaging

Ensuring high-quality imaging is essential for accurate nanoparticle characterization, particularly in the context of NTA measurements. To evaluate the image quality, we conducted a comprehensive evaluation using three complementary approaches: (A) sequential ray tracing simulations with Zemax OpticStudio, (B) experimental NTA measurements with our in-house NanoSight NS300 device, and (C) a comparative analysis with the findings reported by Špačková *et al* ^3^.

1. **Sequential ray tracing analysis using Zemax OpticStudio**

To evaluate the imaging performance of our microscope setup, we used sequential ray tracing in Zemax to calculate the point spread function (PSF) and the ray distribution in the image plane. In detail, sequential ray tracing in Zemax OpticStudio is a method of modelling the propagation of light through a series of ordered optical elements. This approach assumes that rays follow a defined sequence, moving from one optical surface to the next in a predictable, linear fashion. This makes it particularly well suited to the design and optimization of imaging systems where the optical path is well structured, such as cameras, microscopes, telescopes and eyepieces, and is therefore relevant to the microscopic imaging setup used in this work.

1. *Simulation of the used configuration*

The results of the ray tracing simulations performed to reveal the imaging properties of the microscopic arrangement (Fig. S6(a)) are discussed below. Specifically, the PSF (top row of Fig. S7) and the corresponding ray distribution (bottom row of Fig. S7) are computed for three different locations of a light-emitting nanoparticle along the microscopic axis inside the water-filled core of the HCW, considering the configuration shown in Fig. S6(b): The microchannel was approximated as a single polymer membrane with a thickness of 1.5 µm and a refractive index of 1.55 (operation wavelength $\lambda_{0}$ = 532 nm), while the sidewalls and lower membrane were omitted as they are expected to have minimal effect on imaging. Nanoparticle imaging was performed with an Olympus Plan N 10x0.25 infinity corrected objective. The microscope slide was made of borosilicate glass (D263M) with a thickness of 130 µm and a refractive index of 1.526. The distance between the slide and the ARE/waveguide channel was 1.25 mm, filled with water (refractive index of 1.335).


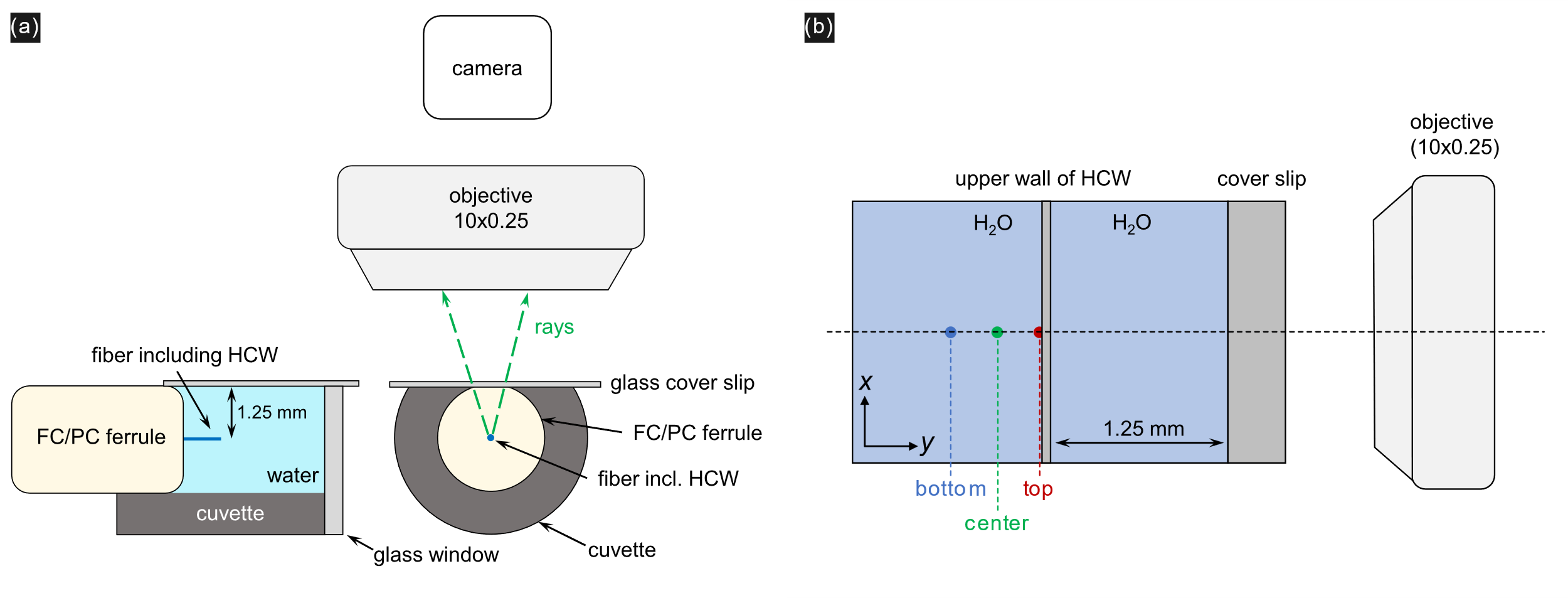


**Fig. S6**: Sketch of the experimental configuration used for the nanoparticle tracking analysis experiments (left: side view of the sample in the water environment; right: position of the mounted sample in the microscope). (b) Sketch of the simulated configurations, including the different materials and interfaces. The three different colored dots indicate the considered positions of the light emitting nanoparticle (blue: bottom position; green: center position inside the waveguide core; red: top position near the top polymer layer). The horizontal black dashed line indicates the axis of the microscope.

For a nanoparticle positioned at the center of the HCW-channel (Fig. S7(a) and green dashed line in Fig. S6(b)), all rays (blue) fall within the Airy disk region (dashed purple circle in Fig. S7(a)), confirming diffraction-limited imaging. This conclusion is supported by the small RMS value of the distribution of the rays (13.4 µm), which is smaller than the Airy disk diameter (26 µm). It is important to note that even in a diffraction-limited system, the inherent diffraction of light prevents an ideal sharp focus where all rays converge to a single point. For the lower position of the nanoparticle (Fig. S7(b) and blue dashed line in Fig. S6(b)), a similar ray distribution is observed, with a slightly higher RMS value (16 µm). For the top position of the nanoparticle close to the polymer membrane (Fig. S7(c) and red dashed line in Fig. S6(b)), a small fraction of rays extend beyond the Airy disk, while the RMS value remains below the Airy disk diameter, confirming diffraction-limited imaging in this case as well.

Overall, these results show that the optical resolution of our system is diffraction-limited, with spherical aberrations being negligible. The only relevant effect is a slight defocusing (c.f. PSFs in the top row of Fig. S7), which is minimal and not relevant for nanoparticle tracking, as only the center of the light distribution is required for accurate tracking, while its extension is of minor importance.


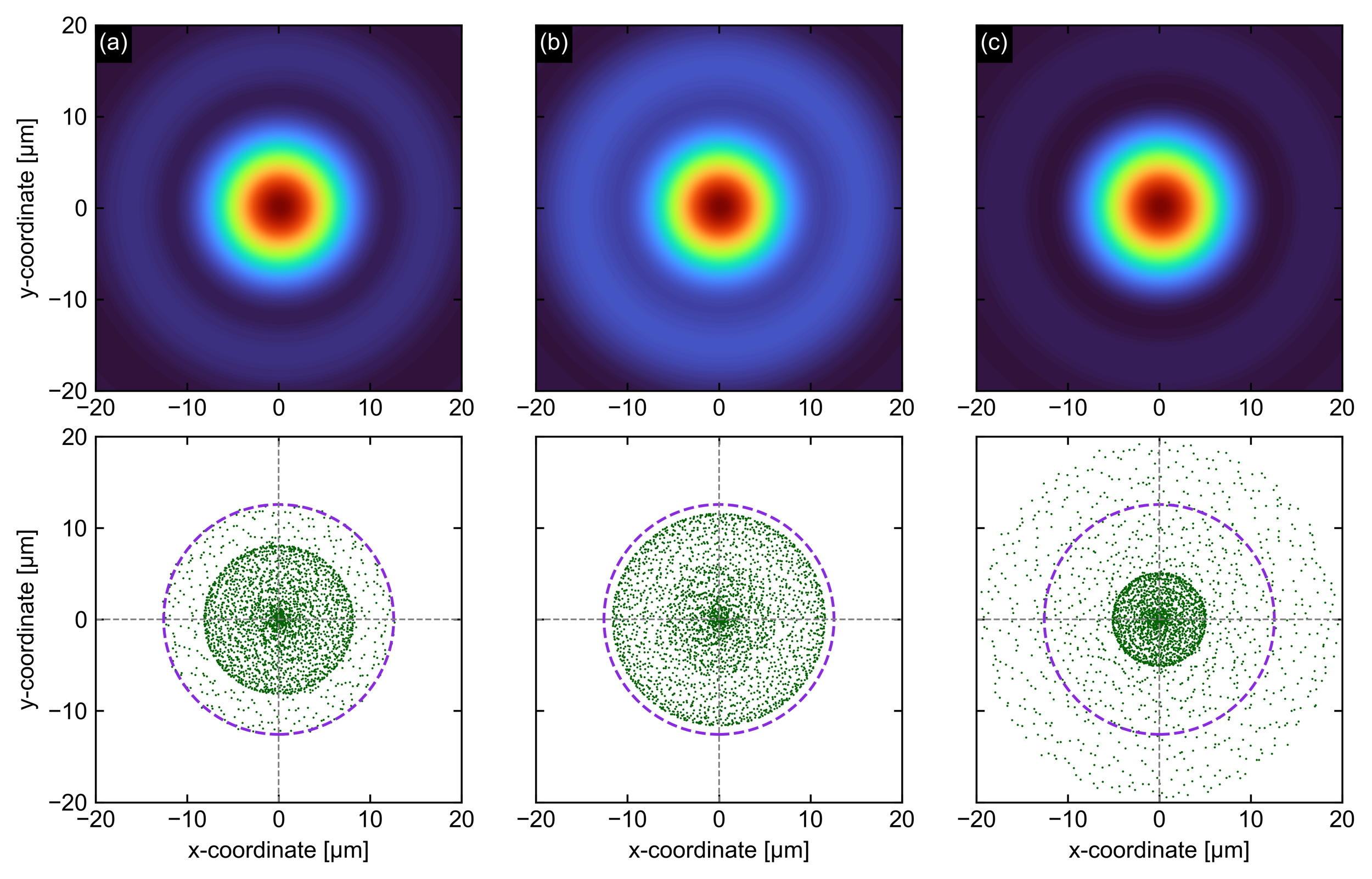


**Fig. S7**: Results of the ray tracing simulations of the three locations of the light emitting nanoparticle inside the waveguide channel shown in Fig. S6(b) ((a): center (green) position, (b): bottom (blue) position, (c): top (red) position). The top row shows the point spread function (linear color scale). The bottom row visualizes the ray distributions in the image plane (each green dot refers to a single ray). The traced rays fill the objective aperture (0.25 NA) with uniform apodization and random distribution. The purple dashed circle represents the Airy disk, which indicates the limit of diffraction (radius $r_{Airy}=1.2197.\lambda_{0}/(2.NA)$). Note that a PSF and the corresponding system are considered diffraction-limited if the root mean square (RMS) of all rays is smaller than the Airy disk diameter (~2.6 μm in the object plane and ~26 μm in the image plane for the optical system considered), indicating that imaging performance is solely governed by diffraction, achieving maximum resolution without degradation from aberrations.

1. *Simulation of the optimized configuration*

In principle, the imaging properties, especially the RMS value of the ray distributions, can be improved by reducing the distance between the cover glass and the objective. To demonstrate this effect, this distance was reduced from 1.25 mm to 0.2 mm and the simulations were repeated (Fig. S8). The results show a qualitatively similar behavior to that shown in Fig. S7, with (i) significantly smaller RMS values, (ii) all rays confined within the Airy disk region, and (iii) a nearly identical PSF in all three cases.


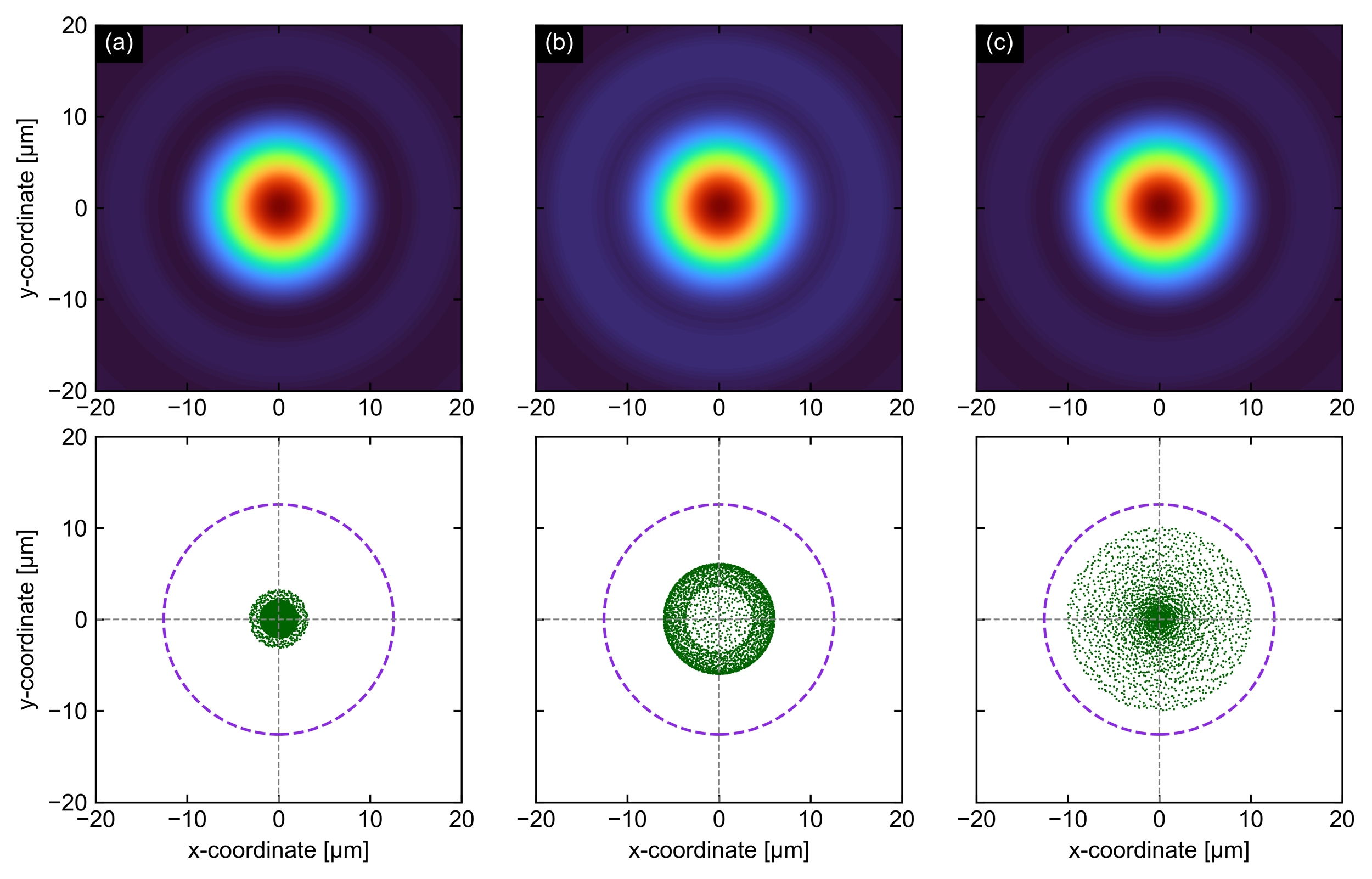


**Fig. S8**: Ray tracing simulations similar to Fig. S7 for a reduced distance between the objective and the cover glass (0.2 mm), considering the three different positions of the light emitting nanoparticle inside the HCW-channel: (a) center (green) position, (b) bottom (blue) position, and (c) top (red) position.

1. *Consideration of tilt of cover glass*

To assess the effect of possible misalignments of the microscopic setup, simulations were repeated assuming a 3° tilt of various elements relative to the microscope axis. In principle, such a tilt could cause the PSF and spot diagram to deviate from their azimuthally symmetric shapes, which should impact the captured images in the tracking experiments.

The simulations show that tilting the HCW had no effect on imaging, due to the symmetric arrangement of water on both sides of the polymer membrane. In contrast to that, tilting the cover glass results in a significant change (Fig. S9), with the PSF shifting towards an asymmetric distribution (top row, Fig. S9). The spot diagram also shows this asymmetry, with some rays falling outside the Airy disk, thus causing aberrations. These results therefore highlight the need for precise alignment of the optical elements prior to experiments to minimize aberrations and ensure optimal imaging quality.


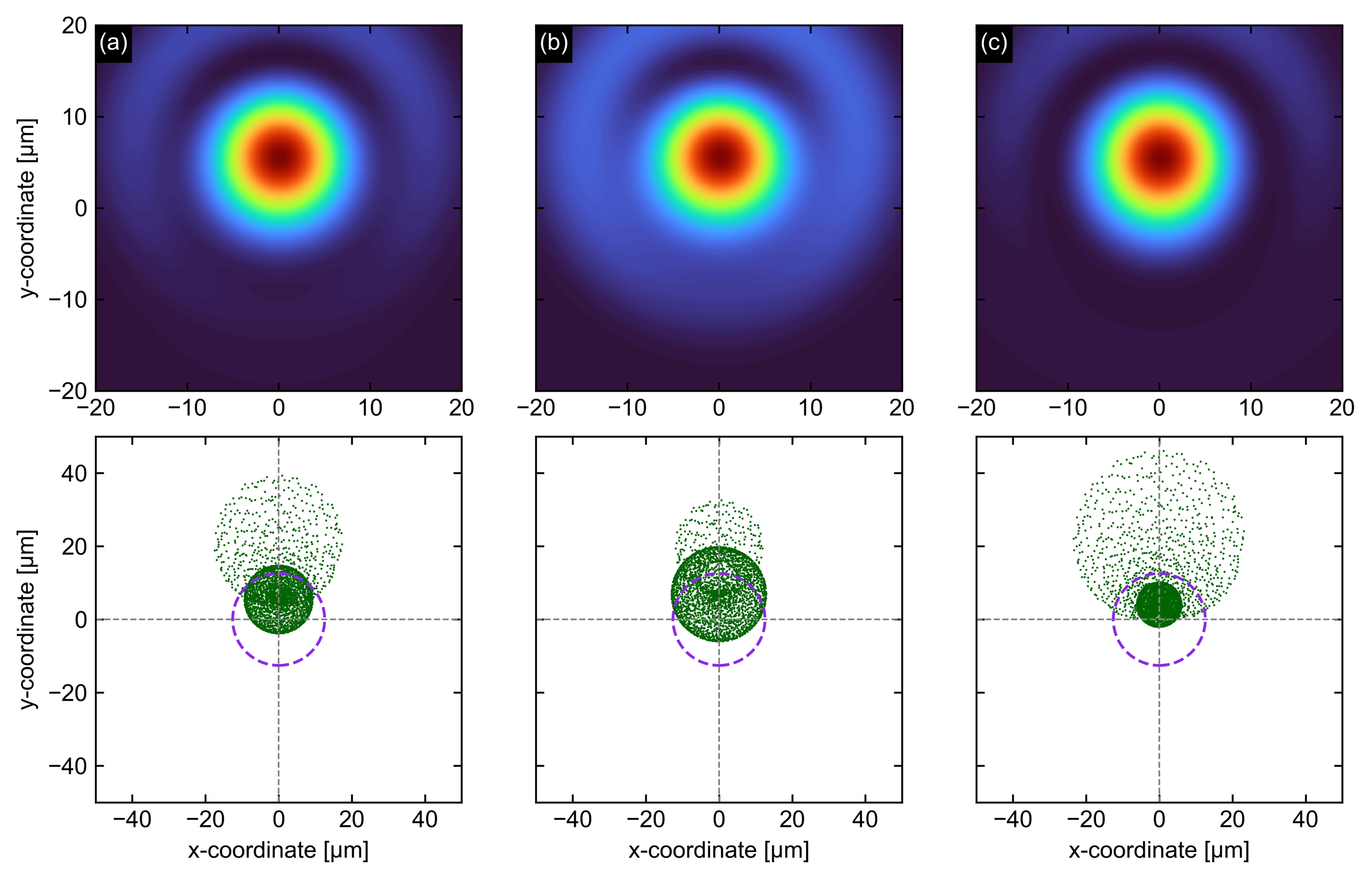


**Fig. S9**: Zemax-based ray tracing simulations including a geometry similar to Fig.S7 and taking into account a tilt of the cover glass (with respect to the microscopic axis) of 3° (distance between the objective and the cover glass: 1.25mm) for the three different positions of the light emitting nanoparticle inside the HCW-channel.

We have also applied this tilt analysis to the above case with a smaller distance between the cover glass and the objective (0.2mm, Fig. S10). Again, deviations from the azimuthal asymmetry can be seen, although these are somewhat smaller than in the case of a larger distance. It can therefore be concluded that, regardless of the configuration used, it is always necessary to achieve optimum alignment prior to the actual tracking experiments in order to minimize the risk of external influence.


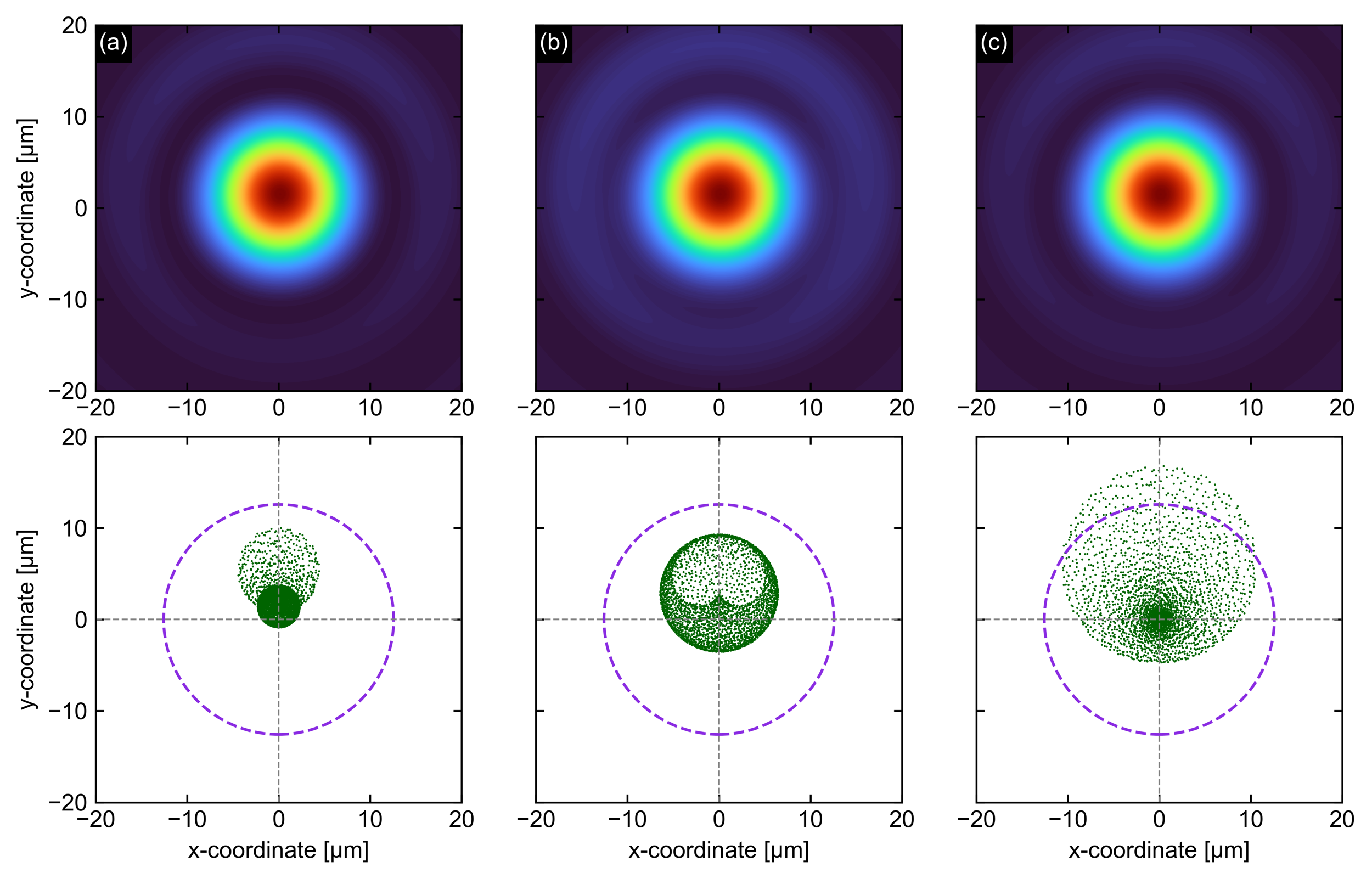


**Fig. S10**: Zemax-based ray tracing simulations performed using a geometry similar to Fig.S7 incorporating a 3° tilt of the cover glass relative to the microscope axis (with a distance of 0.2 mm between the objective and the cover glass) for the three discussed positions of the light emitting nanoparticle within the HCW channel.

1. **Direct comparison of selected frame of our approach and NanoSight**

A direct comparison of the imaging characteristics of our system with those of a commercially available device, the NanoSight NS300, is provided in this section. The NanoSight has a field of view of 100 × 80 × 10 µm and is equipped with a PL 20x0.4 objective. It operates at a wavelength of 488 nm with a recommended particle concentration range of 10^7^-10^9^ particles/mL. Typical measurement times range from 30 to 60 seconds. A key difference between the NanoSight and our system is its significantly lower frame rate of 25 fps, whereas our study used a frame rate of 400 fps, allowing for higher temporal resolution.

To compare the imaging characteristics, additional measurements were performed with the NanoSight device using one of the ensembles from our study (100 nm gold nanoparticles). Selected images from the HCW experiments and the NanoSight measurements are shown in Fig. S11 ((a): HCW-NTA measurements, (b) NanoSight measurements). It should be noted that the images from the NanoSight measurements are those provided directly by the device, and we cannot confirm whether they have undergone any post-processing as we do not have access to the underlying software code.


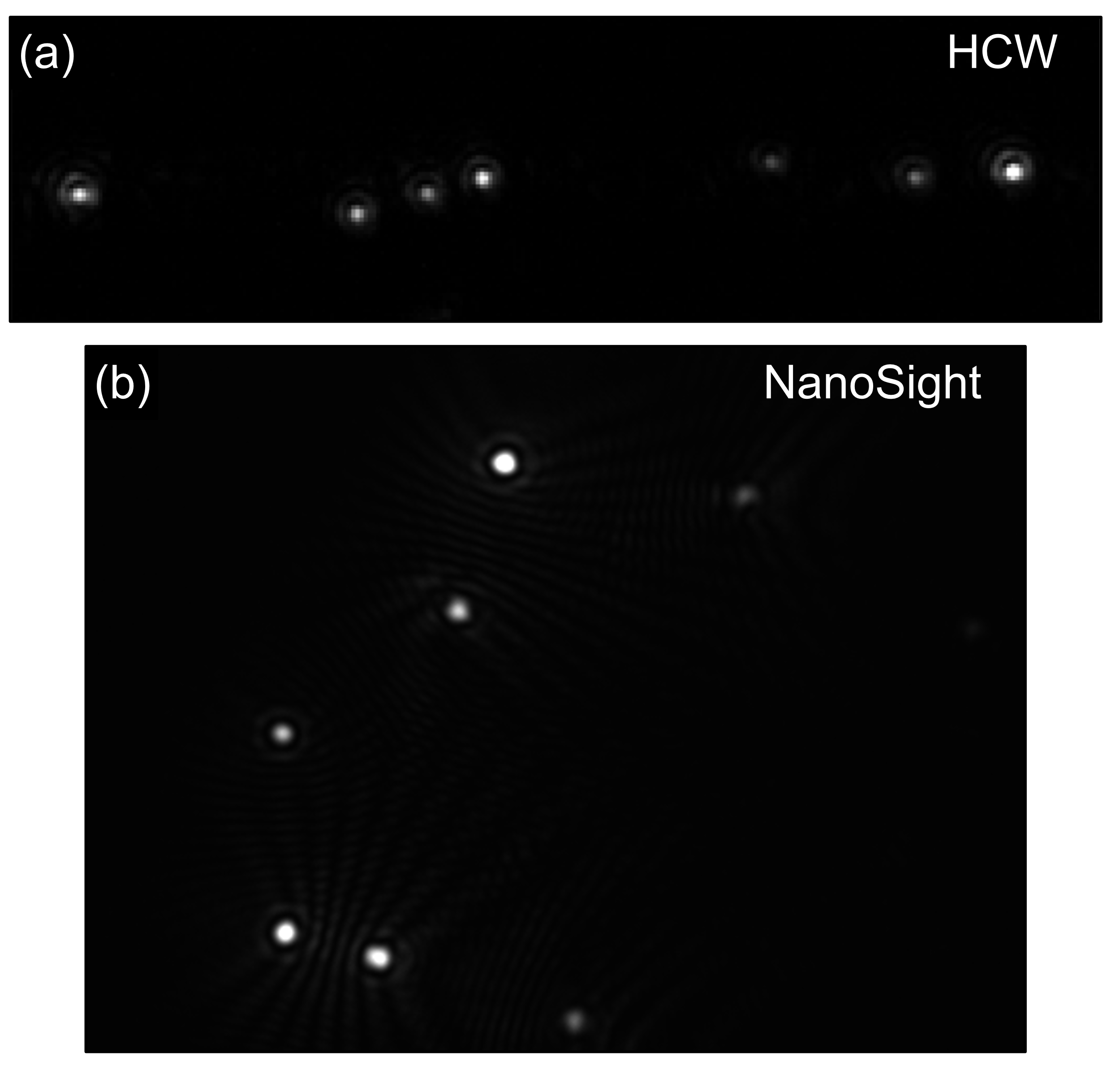


**Fig. S11**: Selected images of diffusing nanoparticles extracted from the recorded movies (nanoparticle ensemble with mean diameter of 100 nm): (a) HCW-based measurements. (b) NanoSight NS300.

The following aspects are visible: Both the HCW-NTA method and the measurements from the NanoSight show pronounced Airy rings around the central light spot, generally reflecting good image quality. The central regions of the nanoparticle light distributions are more azimuthally symmetric in the HCW experiments compared to the NanoSight measurements, which slightly deviate from a circular distribution. As a result, the HCW approach allows a more accurate determination of the center of the light distribution, principally resulting in improved tracking accuracy. Note that the slight asymmetry of the Airy rings in both distributions presumably results from a slight tilt of one of the optical elements involved, as discussed in the ray tracing simulations shown in the previous section. In addition, the light distributions in the NanoSight measurements appear saturated and thus deviate from a Gaussian profile, which can further complicate position determination. In contrast, the HCW measurements were taken with care to avoid saturation and to achieve Gaussian-type intensity distributions. In summary, the HCW-NTA method provides a higher quality image that allows for more accurate position retrieval and thus tracking.

1. **Comparison to the work of Špačková *et al.***

The work of Špačková *et al.* presents Nanofluidic Scattering Microscopy (NMS), a novel label-free technique for real-time imaging of single biomolecules diffusing in a dielectric nanofluidic channel. NSM enables the detection of freely diffusing species, overcoming the limitations of surface binding techniques. The method allows the determination of molecular weight from optical contrast and hydrodynamic radius from diffusivity, providing insight into biomolecular conformational states. The study demonstrates the applicability of NSM by analyzing extracellular vesicles in conditioned cell culture medium.

The results presented in the work are excellent and closely related to our study, thus we believe that the HCW-NTA approach in general can be very valuable in this context. Table S4 summarizes the key features of the mentioned study in comparison to our work.

**Tab. S4:** Comparison of key features of our study with the work of Špačková *et al*.

|  | Our study | Špačková *et al.* |
| --- | --- | --- |
| microscopic setup | commercial dark field microscope (Olympus) | commercial dark field microscope (Mad City Labs RM21) |
| camera | CMOS camera (Basler) | CMOS camera (Andor Zyla) |
| frame rate [fps] | 400 and 600 | 200 |
| max. track length | 1800 | 1500 |
| measurement duration [s] | 10 (600fps), 20 (400fps) | 60 |
| pixel area [pixel] | 1920 x 100 | 30 x 600 and 30 x 2160 |
| NA imaging objective | 0.25 (dry) | 1.49 (immersion oil) |
| magnification | 10x | 100x |
| method | tracking and MSD | tracking and differential imaging |
| photonic platform | fiber-interfaced hollow-core waveguide | silica nanochannel |
| light properties | monochromatic (532nm) | polychromatic (450nm … 750nm) |

Regarding imaging quality, it is noteworthy that the work of Špačková et al. does not explicitly provide a selected image of a tracked frame, making a direct comparison of the image quality between the two approaches difficult. However, as the work of Špačková *et al*. relies on a commercially available high-performance dark-field microscope (Mad City Labs RM21), we strongly believe that their results include a very high image quality.

An important difference between our work and that of Špačková *et al*. is the use of monochromatic light in our setup compared to their use of polychromatic light. We believe that polychromatic light may offer advantages such as reduced speckle and interference, and this may be an area of research that we will consider in the future. Furthermore, our experiments use a microscope objective with a comparatively small numerical aperture (NA = 0.25) to image the particles, whereas the Špačková *et al.* use an immersion objective with NA = 1.49. While the latter allows imaging of smaller particles due to high magnification (>200), it is significantly more expensive than the objective used in our setup. In summary, it can be said that both methods have advantages and disadvantages depending on the application and circumstances.

Sec. S9: Comparative analysis of NTA tracking length

The tracking length in NTA significantly influences the precision of particle size and diffusion measurements. The data processing of NTA is often based on mean square displacement (MSD) analysis of the diffusive motion of individual nanoparticles. This statistical approach determines the diffusion coefficient of the single nanoparticles, which can then be translated into hydrodynamic diameter using the Einstein-Stokes relation. Due to its statistical nature, the accuracy of the diffusion coefficient determination improves with longer observation times, i.e. longer tracks. This relationship is described by the Cramer-Rao lower bound, which relates the standard deviation of the determined diffusion coefficient $\sigma_{d}$ or hydrodynamic diameter to the number of frames per track $N$

$\begin{aligned} \frac{\sigma_{d}}{D}\geq\sqrt{\frac{2}{N-1}\left( 1+2\sqrt{1+2x} \right)}\approx\sqrt{\frac{2}{N-1}}\#\left( S2 \right) \end{aligned}$

with the localization error $x$, which is negligible for FaNTA ($x\ll1$)^4^. This expression clearly shows that longer tracks (i.e. larger values of $N$) allow diameters to be determined with greater accuracy.

Given the importance of long tracking lengths, a literature analysis will be conducted to evaluate and compare our results. To address this, we will (A) present and discuss the key performance indicators summarized from published NTA-related studies on trajectory length, (B) discuss the key features of the NanoSight NS300, and (C) address the results presented in the study of Špačková *et al.*

1. **Broad literature comparison**

In the experiments reported in this work, we obtain (for the 100 nm ensemble) a maximum trajectory length of 1800, which is in the regime of other works, as shown in Tab. S5 which compares several NTA-related studies reported in the literature. Note that although the maximum number of frames obtained in our work is in the same range as the studies shown, our work operates at a substantially higher frame rate of (400 fps compared to 25-30fps), thus allowing for a higher overall temporal resolution.

**Tab. S5:** Comprehensive summary of Nanoparticle Tracking Analysis (NTA) studies in the context of trajectory related parameters.

| name of paper | frame rate (fps) | max. no. frames | measurem. duration [s] | Ref. |
| --- | --- | --- | --- | --- |
| Nanomotor Tracking Experiments at the Edge of Reproducibility | 25 | 1500 | 60 | ^[5]^ |
| High-Resolution Nanoparticle Sizing with Maximum A Posteriori NTA | 25 | Not specified | Not specified | ^[6]^ |
| Development of a New Methodology to Determine Size Differences in Nanoparticles | 30 | 1800 | 60 | ^[7]^ |
| Evaluation of NTA for Total Virus Particle Determination | 30 | 1800 | 60 | ^[8]^ |
| Critical Evaluation of Nanoparticle Tracking Analysis | 30 | 1200 | 40 | ^[9]^ |
| Sizing and Phenotyping of Cellular Vesicles Using NTA | 30 | 1800 | 60 | ^[10]^ |
| NTA Monitors Microvesicle and Exosome Secretion from Immune Cells | 30 | 1800 | 60 | ^[11]^ |

1. **Comparison to commercial device**

Below we present a comparison of our system with the NanoSight NS300 in the context of track length. A detailed examination of the exported data from our NanoSight NS300 measurements revealed that the maximum track length achieved with this instrument is consistently capped at 100, with no longer tracks appearing. This observation is at least for us unexpected since, as previously discussed, longer tracks should result in greater accuracy. As the details of the data evaluation process and the software used are not accessible, we cannot determine the reason for this limitation of the NS300. A possible explanation could be the low frame rate of 25 fps, which may limit the track length as particles may diffuse out of the field of view, potentially causing a loss of trajectory, or an incomplete trajectory linking. However, as this is speculative, we have decided not to investigate this further and will not include a direct comparison with the NanoSight system in the context of trajectory length.

1. **Comparison to the work of Špačková *et al.***

A direct comparison of the properties of our system with those reported by Špačková *et al.* in terms of trajectory length is shown in Tab. S4. The study presented by Špačková *et al.* describes a system operating at a frame rate of 200 fps, with a maximum trajectory length of 1500 frames and a measurement duration of 60 seconds per video. In comparison, our system achieves a higher frame rate of 400 fps and even 600fps, principally providing a higher temporal resolution. Our system supports a maximum track length of 1800 frames, which is in the same order of magnitude as the referenced study, thus providing the same ability to track particles over long periods of time. The maximum measurement duration of our system is 20 seconds, which is shorter than the 60 seconds reported in the aforementioned study. While this may limit the observation of slower dynamics, our system is capable of capturing fast processes with high precision.

Sec. S10: Performance comparison between HCW-NTA and NanoSight NS300

1. **Measurement of the nanoparticle ensemble with an average diameter of 50 nm**

Multiple measurements of the 50 nm nanoparticle ensemble in water were conducted using the NS300. For that, stock solutions were diluted 400 times to achieve a particle concentration of 10^8^ NP/mL, which is within the specified range of the NS300. Measurements were performed at ambient conditions of 20°C, leading to a dynamic viscosity of 1 mPas. Note that the NS300 does not allow adjusting the frame rate (fixed to 25 fps) and limits the measurement duration to 60 seconds, resulting in a maximum of 1500 frames per trajectory. Five consecutive measurements were taken and the results are summarized in Tab. S6.

**Tab. S6:** Results of measurements of the ensemble of nanoparticles with a mean diameter of 50 nm obtained with the NanoSight NS300 instrument.

|  | **M1** | **M2** | **M3** | **M4** | **M5** | **mean** |
| --- | --- | --- | --- | --- | --- | --- |
| mean hyd. diam. [nm] | 58.2 | 55.5 | 58.5 | 56.4 | 55.8 | 56.9 ±1.4 |
| standard deviation [nm] | 13.7 | 3.0 | 12.9 | 8.4 | 3.8 | 8.4 ± 5 |
| total trajectories [frames] | 1341 | 1267 | 1304 | 1300 | 1481 | 1338 |
| valid trajectories [frames] | 566 | 531 | 537 | 524 | 633 | 558 |
| particles/frames | 10 | 10 | 11 | 9 | 11 | 10 |
| particle concentration (10^7^NPs/mL) | 9.79 | 9.39 | 9.64 | 8.28 | 10.7 | 9.56 |

Inspection of Tab. S6 shows that a significant proportion of tracks (over 50%) are invalid and excluded, which is much higher than in HCW-NTA and is likely to reduce accuracy. NanoSight uses a threshold that removes all tracks shorter than 5 frames, which is significantly shorter than in HCW-NTA. The measured mean hydrodynamic diameter agrees well with both our value and the expected value, while the DLS measurement shows a bias towards larger diameters, confirming that NTA is highly suitable for nanoparticle characterization. Notably, the five measurements show considerable variation in standard deviation of the hydrodynamic diameter. In particular, M2 and M5 are very close to our measurements and have small standard deviations, whereas M1, M3 and M4 have significantly larger diameters and higher standard deviations. This variation may be due to the small number of frames per track, as the NS300 consistently capped trajectories at a length of 100, combined with the low frame rate of 25 fps. The concentration determined is accurate and within the expected range.

1. **Comparison of HCW and NS300**

This comparison of the two approaches shows that, despite significant differences in system parameters, both techniques can accurately determine the mean hydrodynamic diameter of nanoparticle ensembles. In particular, our technique operates at a minimum frame rate of 400 fps, whereas the NanoSight NS300 has a fixed frame rate of 25 fps and limits all trajectories to a maximum length of 100 frames. As a result, HCW-NTA is better suited to monitoring fast dynamic processes that cannot be resolved at the 25 fps frame rate of the NS300. In addition, the number of invalid tracks in the NanoSight measurements is significantly higher than in the HCW-NTA measurements, potentially providing better statistical significance with the HCW-NTA instrument. However, we refrain from extending the discussion to the details of data analysis as we do not have access to the NS300 software and any speculation in this regard would be purely hypothetical.

References

1. Deng, A. et al. Analyzing mode index mismatch and field overlap for light guidance in negative-curvature fibers. *Optics Express* **28**, 27974–27988 (2020).

2. Dechadilok, P. & Deen, W. M. Hindrance Factors for Diffusion and Convection in Pores. *Industrial & Engineering Chemistry Research* **45**, 6953–6959 (2006).

3. Špačková, B. et al. Label-free nanofluidic scattering microscopy of size and mass of single diffusing molecules and nanoparticles. *Nature Methods* **19**, 751–758 (2022).

4. Nissen, M. et al*.* Nanoparticle Tracking in Single-Antiresonant-Element Fiber for High-Precision Size Distribution Analysis of Mono- and Polydisperse Samples. *Small* **18**, 2202024 (2022).

5. Novotný, F. & Pumera, M. Nanomotor tracking experiments at the edge of reproducibility. *Scientific Reports* **9**, 13222 (2019).

6. Silmore, K. S. et al. High-Resolution Nanoparticle Sizing with Maximum A Posteriori Nanoparticle Tracking Analysis. *ACS Nano* **13**, 3940–3952 (2019).

7. Pellequer, Y. et al*.* Development of a new methodology to determine size differences of nanoparticles with nanoparticle tracking analysis. *Applied Nanoscience* **11**, 2129–2141 (2021).

8. Kramberger, P. et al. Evaluation of nanoparticle tracking analysis for total virus particle determination. *Virology Journal* **9**, 265 (2012).

9. Filipe, V.et al. Critical Evaluation of Nanoparticle Tracking Analysis (NTA) by NanoSight for the Measurement of Nanoparticles and Protein Aggregates. *Pharmaceutical Research* **27**, 796–810 (2010).

10. Dragovic, R. A. et al*.* Sizing and phenotyping of cellular vesicles using Nanoparticle Tracking Analysis. *Nanomedicine: Nanotechnology, Biology and Medicine* **7**, 780–788 (2011).

11. Soo, C. Y. et al*.* Nanoparticle tracking analysis monitors microvesicle and exosome secretion from immune cells. *Immunology* **136**, 192–197 (2012).
